# Inflammation-Driven Downregulation of CYP2E1 Is Associated with Attenuated Diethylnitrosamine (DEN)-Induced Hepatocarcinogenesis

**DOI:** 10.3390/cells15060546

**Published:** 2026-03-19

**Authors:** Yoshihiro Tsuchiya, Yusuke Sotomaru, Akinori Kanai, Shin Maeda, Hideaki Kamata

**Affiliations:** 1 Department of Nutrition and Health, Faculty of Human Life Studies, Hiroshima Jogakuin University, Hiroshima 732-0063, Japan; 2 Department of Molecular Medical Science, Graduate School of Biomedical Science, Hiroshima University, Hiroshima 734-8553, Japan; 3 Natural Science Center for Basic Research and Development, Graduate School of Biomedical and Health Sciences, Hiroshima University, Hiroshima 734-8553, Japan; sotomaru@hiroshima-u.ac.jp; 4 Graduate School of Frontier Sciences, The University of Tokyo, Chiba 277-8562, Japan; akkanai@edu.k.u-tokyo.ac.jp; 5 Department of Gastroenterology, School of Medicine, Yokohama City University, Yokohama 236-0004, Japan; smaeda@med.yokohama-cu.ac.jp

**Keywords:** hepatocellular carcinoma, diethylnitrosamine, CYP2E1, IKKβ/NF-κB, hepatic xenobiotic metabolism

## Abstract

**Highlights:**

**What are the main findings?**

- Transgenic (Tg)-IKKβΔhep mice develop spontaneous chronic hepatitis and fibrosis yet are resistant to DEN-induced hepatocarcinogenesis.
- Chronic inflammatory signaling suppresses pericentral CYP2E1 and broadly down-regulates hepatic xenobiotic-metabolism programs.

**What are the implications of the main findings?**

- Attenuated HNF4α–PXR–CAR transcriptional output is associated with reduced DEN-triggered DNA-damage responses and p53 activation.
- These data support an inflammation-driven “metabolic gatekeeping” model in which chronic liver injury can constrain chemical tumor initiation by suppressing procarcinogen-metabolizing capacity.

**Abstract:**

Inflammation is widely viewed as a driver of hepatocellular carcinoma (HCC), yet inflammatory signaling also reshapes hepatic xenobiotic metabolism. Here, we established transgenic (Tg) IKKβ^Δhep^ mice (Tg-IKKβ^Δhep^), which combine hepatocyte-specific IKKβ deletion with liver expression of a nuclear, kinase-inactive IKKβ mutant (NLS-IKKβKN). Tg-IKKβ^Δhep^ mice developed spontaneous chronic hepatitis and progressive fibrosis but were strikingly resistant to diethylnitrosamine (DEN)-induced hepatocarcinogenesis, with markedly reduced tumor multiplicity and total tumor burden. Despite persistent inflammatory injury, DEN-triggered oxidative DNA damage and p53 activation were markedly attenuated, compatible with reduced tumor initiation. Transcriptomic and biochemical analyses revealed broad repression of xenobiotic-metabolizing cytochrome P450 genes, including the pericentral enzyme CYP2E1, accompanied by reduced CYP2E1 protein abundance. This was associated with impaired HNF4α–PXR–CAR transcriptional output and reduced HNF4α occupancy at target promoters. Acute TNFα or IL-1β exposure recapitulated this repression, in part through reduced PGC-1α expression and decreased RNA polymerase II recruitment to target promoters. In parallel, pericentral xenobiotic metabolism was blunted, a change that could plausibly diminish DEN bioactivation and genotoxic stress. Together, these findings support a “metabolic gatekeeping” model in which chronic inflammation can constrain chemical hepatocarcinogenesis by attenuating carcinogen-metabolizing capacity.

## 1. Introduction

Hepatocellular carcinoma (HCC) is the most prevalent histological subtype of primary liver cancer and remains a leading cause of cancer-related mortality worldwide [1,2,3,4]. HCC typically develops on a background of chronic liver injury and inflammation that progresses to cirrhosis, driven by hepatitis virus infection, alcohol-associated liver disease, and metabolic dysfunction-associated steatohepatitis (MASH; previously termed NASH) [2,3,4,5,6]. Despite therapeutic advances, the molecular mechanisms linking chronic hepatitis to tumor initiation and progression remain incompletely understood [3,4].

The liver displays characteristic metabolic zonation within the hepatic acinus, consisting of periportal, intermediary, and pericentral regions [7,8,9,10,11]. Periportal hepatocytes preferentially execute β-oxidation and gluconeogenesis, whereas pericentral hepatocytes are enriched in glycolytic and xenobiotic-metabolizing enzymes, particularly cytochrome P450 (CYP) isoforms capable of bioactivating diverse procarcinogens [7,8,9,10,11]. Consistent with these functional specializations, carcinogenic insults can initiate tumorigenesis from differentiated hepatocytes (including pericentral hepatocyte populations), generating progenitor-like states that may ultimately progress to HCC [11,12]. Diethylnitrosamine (DEN), a prototypic chemical hepatocarcinogenesis model, is metabolically activated predominantly in pericentral hepatocytes by CYP2E1, generating DNA-reactive intermediates that induce genotoxic stress (DNA damage) while also promoting hepatocyte injury and cell death [13,14,15].

Hepatocyte injury and death promote the release of damage-associated molecular patterns (DAMPs), thereby activating liver macrophages (including Kupffer cells) and inducing production of mitogens such as IL-6 and EGFR ligands that drive compensatory proliferation [16,17,18]. While compensatory proliferation is essential for tissue repair and homeostasis, under carcinogenic conditions it can permit clonal expansion of DNA-damaged hepatocytes and accelerate liver tumorigenesis [16,17,18]. Moreover, macrophage-derived cues can attenuate p53-dependent genome surveillance in DNA-damaged hepatocytes via CD44-dependent proliferative signaling, enabling evasion from p53-driven apoptosis or senescence [19]. Thus, tumor initiation can be viewed as a coordinated process involving procarcinogen bioactivation, hepatocyte injury, compensatory proliferation, and escape from genome surveillance.

The IκB kinase (IKK)/NF-κB pathway is a central regulator of inflammation and cell survival and exhibits context-dependent, bidirectional functions in liver disease and HCC [20,21,22,23,24,25,26]. Hepatocyte IKKβ/NF-κB signaling contributes to intracellular redox homeostasis and oxidative stress control [21,22]. Hepatocyte-specific deletion of IKKβ enhances ROS accumulation and hepatocyte death and promotes DEN-induced hepatocarcinogenesis through compensatory proliferation [20,21,22]. In addition, deletion of NEMO/IKKγ in liver parenchymal cells causes spontaneous steatohepatitis and HCC, supporting a tumor-suppressive role of hepatocyte NF-κB signaling in multiple settings [23,24,25]. Conversely, inhibition of hepatocyte NF-κB can delay tumor progression in inflammation-driven models, and in lymphotoxin-dependent settings hepatocyte IKKβ deficiency can suppress hepatitis and tumor formation, suggesting that its function can switch depending on etiology, cellular compartment, and the stage of carcinogenesis [26].

However, it remains unclear how hepatocyte IKKβ/NF-κB signaling regulates xenobiotic metabolic competence, particularly CYP2E1-dependent procarcinogen bioactivation, and how this regulation influences subsequent DNA damage and genome surveillance responses. In our prior work, we showed in cultured cells that nuclear IKKβ promotes IκBα ubiquitination and degradation under stress conditions such as ultraviolet irradiation, thereby activating NF-κB while concomitantly contributing to cell death responses [27]. Whether this nuclear IKKβ signaling axis operates in vivo and contributes to liver pathophysiology, however, has not been determined.

To extend these observations in vivo and to disentangle kinase-activity-dependent versus kinase-activity-independent functions of IKKβ, we employed a nuclear-localized, kinase-dead IKKβ mutant (NLS-IKKβKN). This approach was designed to independently validate our prior report that ultraviolet irradiation can activate NF-κB without canonical IKK activation, in which nuclear IKKβ acts as an adaptor to promote IκBα ubiquitination and degradation (Tsuchiya et al., 2010) [27], and to test whether this non-canonical nuclear IKKβ axis influences hepatic xenobiotic metabolism and carcinogen-induced genotoxic stress.

To address these questions, we generated a novel mouse model that spontaneously develops chronic hepatitis and cirrhosis-like pathology by expressing an IKKβ mutant allele in the background of hepatocyte-specific IKKβ deficiency. Interestingly, these mice showed strong resistance to DEN-induced HCC. This phenotype was associated with marked downregulation of CYP2E1 and disruption of the HNF4α–PXR–CAR nuclear receptor network that orchestrates hepatic xenobiotic metabolism [28,29]. Because inflammatory signaling is widely recognized to alter hepatic CYP gene programs and drug metabolism [30,31], our findings propose a context-dependent protective mechanism in which altered IKKβ signaling may dampen procarcinogen bioactivation and oxidative DNA damage during chemical hepatocarcinogenesis.

## 2. Materials and Methods

### 2.1. Animals and Experimental Design

Animal experiments were conducted in accordance with the institutional guidelines of Hiroshima University and applicable Japanese regulations for the care and use of laboratory animals, and are reported in compliance with the ARRIVE 2.0 guidelines. The study protocol was reviewed and approved by the Hiroshima University Animal Experiment Committee (approval no. A17-154; approved on 24 December 2019). In accordance with the 3R principles (Replacement, Reduction, and Refinement), animal use was limited to experiments requiring in vivo models, group sizes were kept to the minimum necessary to ensure robust analyses, and animal welfare monitoring was performed throughout the study.

Mice were housed in a specific pathogen-free (SPF) animal facility under controlled environmental conditions (22 ± 2 °C, 50 ± 10% humidity, 12 h light/12 h dark cycle). Animals were provided standard chow and water ad libitum. Mice were housed in individually ventilated cages (IVCs) with autoclaved paper-based bedding and environmental enrichment (nesting material and shelters). Unless required by experimental conditions, 3–5 mice were housed per cage.

IKKβ-flox/flox (IKKβ^F/F^) mice (harboring loxP-flanked Ikbkb alleles; originally established in the laboratory of Michael Karin (University of California San Diego, La Jolla, CA, USA)) and Albumin-Cre transgenic mice were kindly provided by Professor Shin Maeda (Yokohama City University, Kanagawa, Japan). Hepatocyte-specific IKKβ-deficient mice (IKKβ^Δhep^) were generated by crossing IKKβ^F/F^ mice with Albumin-Cre mice as previously reported [16]. IKKβ^F/F^; Alb-Cre^+^ mice were defined as IKKβ^Δhep^, and Cre-negative littermates (IKKβ^F/F^; Alb-Cre^−^) were used as wild-type-equivalent controls.

To generate mice expressing a nuclear localization signal (NLS)-fused kinase-dead IKKβ mutant, we established transgenic mice carrying an Ikbkb promoter-driven expression cassette. The transgene was constructed in a pRK-based plasmid backbone and comprised a ~4.0 kb mouse Ikbkb 5′ regulatory region (from −4000 to −1 relative to the translation start codon, including non-coding exon(s)/intron(s)), followed by sequences encoding an N-terminal FLAG tag, an NLS, mouse IKKβ (Ikbkb) carrying the kinase-inactivating K44A mutation, and a polyadenylation signal. The expression cassette was introduced into fertilized eggs derived from the IKKβ^F/F^ line by pronuclear injection, resulting in random genomic integration; the genomic insertion site(s) and transgene copy number were not determined. Transgene-positive mice were defined as Tg. Tg-IKKβ^Δhep^ mice were generated by crossing Tg mice with IKKβ^Δhep^ mice, and offspring with the genotype Tg; IKKβ^F/F^; Alb-Cre^+^ were defined as Tg-IKKβ^Δhep^. Depending on the experiment, the following groups were compared: IKKβ^F/F^ (control; IKKβ^F/F^; Alb-Cre^−^), IKKβ^Δhep^ (IKKβ^F/F^; Alb-Cre^+^), Tg (Tg; IKKβ^F/F^; Alb-Cre^−^), and Tg-IKKβ^Δhep^ (Tg; IKKβ^F/F^; Alb-Cre^+^). The transgene design and breeding strategy are shown in Supplementary Figure S1.

Unless otherwise specified, the age and sex of mice at the time of analysis are provided in the corresponding figure legends and/or the relevant Methods subsections. The sex, age (or developmental stage), genotype, and treatment conditions for each cohort are indicated in the relevant Results panels and figure legends.

Genotyping was performed by PCR using genomic DNA isolated from tail biopsies to detect the floxed Ikbkb allele, the Alb-Cre transgene, and the transgene (Tg) allele. Primer sequences and expected amplicon sizes are listed in Supplementary Table S1. Transgene expression in liver tissue was confirmed by immunoblotting using anti-FLAG and anti-IKKβ antibodies (Supplementary Figure S1C,D). Tg-IKKβ^Δhep^ mice were established from multiple founder lines (#3, #6, #9, and #12). Nuclear and cytosolic fractions from mouse liver tissues were prepared using the Nuclear/Cytosol Fractionation Kit (BioVision) according to the manufacturer’s instructions. Fraction purity was verified by immunoblotting for α-Tubulin (cytosolic marker) and Lamin B1 (nuclear marker). Unless otherwise indicated, analyses in this study were performed primarily using the founder #9-derived line.

In vivo treatment protocols (including DEN administration and, where applicable, inflammatory stimuli) are described in the relevant Methods subsections below, including route of administration, dose, timing, and tissue collection time points. These treatments were designed to compare genotype-dependent differences in liver injury, xenobiotic metabolism, and hepatocarcinogenesis under basal and chemically challenged conditions.

Mice were euthanized in accordance with ethical guidelines by CO_2_ inhalation followed by a secondary physical method (cervical dislocation when required). Animals were monitored daily throughout the study for general condition and signs of pain or distress (reduced activity, ruffled fur, abnormal posture, impaired grooming, and rapid weight loss). Humane endpoints were predefined as severe lethargy, persistent inability to access food/water, or other signs of severe distress, and any animal meeting these criteria was promptly euthanized in accordance with the approved protocol. No survival surgery was performed in this study; therefore, postoperative analgesia was not applicable under the approved protocol. No unexpected adverse events were observed.

Study design: This study compared outcomes among genetically modified mouse lines (IKKβ^F/F^, IKKβ^Δhep^, Tg, and Tg-IKKβ^Δhep^) under basal conditions and after chemical injury/carcinogen exposure. Unless otherwise specified, the experimental unit was one individual mouse (1 mouse = 1 biological replicate).

Sample size: No a priori power calculation was performed. Sample sizes were determined based on conventions in similar mouse studies and experimental feasibility, and the exact *n* values are provided in the corresponding figure legends.

Inclusion and exclusion criteria: Mice that completed the planned treatment schedule and tissue collection were included in the analyses. Exclusion criteria were limited to unexpected illness unrelated to the experimental protocol, death before the planned endpoint, or technical failure during sample collection/processing. Unless otherwise stated in the figure legends, no animals or data points were excluded.

Randomization: Randomization was not applied to comparisons between groups defined by genotype. However, to minimize potential confounding, age and sex were matched across groups. In treatment experiments, mice were assigned to intervention groups in a balanced manner within each genotype. In addition, dosing order and measurement order were adjusted to avoid group-wise bias.

Blinding: Investigators were aware of genotype/treatment allocation during animal handling and DEN administration. Outcome assessment was performed in a blinded manner where feasible (e.g., histological evaluation and image-based quantification were conducted using coded samples). Statistical analyses were conducted on coded datasets, and group identities were revealed after completion of the primary analyses.

Outcome measures: The primary outcome measures were tumor incidence and tumor burden at the study endpoint after DEN administration (including tumor number and maximum tumor diameter), as well as serum ALT activity as an index of liver injury. Secondary outcome measures, as described in each experimental section, included histological and immunostaining-based indices, RT-qPCR-based gene expression analyses, and ChIP-based promoter occupancy analyses. Details of statistical analyses are provided in Section 2.15.

Protocol registration: No study protocol was preregistered for this study.

### 2.2. Chemical Hepatocarcinogenesis by DEN in Juvenile Mice (P15, Single Dose)

To induce hepatocellular carcinoma (HCC), diethylnitrosamine (DEN; FUJIFILM Wako Pure Chemical Corporation, Osaka, Japan) was administered once by intraperitoneal injection (25 mg/kg) to 15-day-old male pups, and tumor formation was evaluated 10 months later, as previously described [13,16,20]. After sacrifice, livers were excised and macroscopically inspected on both the surface and cut sections. Nodules with a maximum diameter ≥ 1 mm were counted as tumors, and maximal tumor diameter was recorded when appropriate. Representative liver regions were fixed in 10% neutral-buffered formalin, paraffin-embedded, sectioned, and subjected to H&E staining and other histological analyses as indicated.

### 2.3. Juvenile CCl_4_ Pre-Treatment Followed by DEN (P14 CCl_4_ → P15 DEN)

Male C57BL/6J mice (CLEA Japan, Inc., Tokyo, Japan) were used in this experiment. To assess chemical hepatocarcinogenesis on the background of juvenile liver injury, carbon tetrachloride (CCl_4_) was diluted to 10% (*v*/*v*) in corn oil and administered once by intraperitoneal injection (5 μL/g body weight) to 14-day-old male pups, following established CCl_4_ injury paradigms with minor modifications [32]. The next day (P15), DEN was administered once by intraperitoneal injection (25 mg/kg), and tumor formation was evaluated 10 months later as described in Section 2.2 [16]. Tumors were counted using the same criteria (≥1 mm in maximal diameter).

### 2.4. Chronic CCl_4_-Induced Liver Injury

For chronic liver injury, 8-week-old maleC57BL/6J mice (CLEA Japan, Inc.) received CCl_4_ diluted to 10% (*v*/*v*) in corn oil by intraperitoneal injection (5 μL/g body weight) twice per week for 5 weeks as described previously [32]. Control mice received corn oil alone on the same schedule. Body weight and general condition were monitored throughout the regimen. At the end of the regimen, serum and tissues were collected for biochemical, histological, and molecular analyses.

### 2.5. Acute DEN Challenge (48 h) and DNA Damage Response

For acute assessment of DEN-induced DNA damage responses, 8-week-old mice of each genotype were administered DEN once by intraperitoneal injection (50 mg/kg) and euthanized 48 h later for blood collection and liver harvest [16]. Serum ALT activity was measured, and p21 (Cdkn1a) expression was quantified by RT-qPCR. In addition, immunostaining and immunoblot analyses were performed for markers including phosphorylated H2AX (Ser139).

To assess responses after chronic liver injury, mice subjected to the chronic CCl_4_ regimen (Section 2.4) were allowed a 7-day recovery period and then challenged with DEN (Wako; 50 mg/kg, i.p. once). Livers were harvested 48 h later for RNA extraction and RT-qPCR analysis of p53 target genes (p21 and Bax) as well as Cyp2e1, Hnf4a, and related transcripts [32].

For long-term tumor assessment after chronic injury, mice were treated with DEN following the chronic CCl_4_ regimen as above and were sacrificed 10 months later for analysis of tumor number and tumor size. Four groups were analyzed as appropriate: untreated, DEN alone, CCl_4_ alone, and chronic CCl_4_ followed by DEN.

### 2.6. Acute Cytokine Stimulation (TNFα/IL-1β)

For acute inflammatory stimulation, 6–8-week-old male C57BL/6J mice (CLEA Japan, Inc.) were administered saline (vehicle), mouse TNFα, or mouse IL-1β (BioLegend, San Diego, CA, USA) once by intravenous injection (7.5 μg/kg) and euthanized 4, or after injection for downstream analyses.

### 2.7. Serum Biochemistry

Serum alanine aminotransferase (ALT) activities were determined using the Transaminase CII Test Kit (FUJIFILM Wako Pure Chemical Corp., Osaka, Japan) according to the manufacturer’s protocol.

### 2.8. Immunohistochemistry and Immunofluorescence

Liver tissues were fixed in 10% neutral-buffered formalin, dehydrated through graded ethanol and xylene, and embedded in paraffin. Sections were deparaffinized and rehydrated, followed by heat-induced antigen retrieval in citrate buffer. For immunofluorescence staining, sections were permeabilized with 0.1% Triton X-100 when required.

For immunohistochemistry (IHC), sections were incubated with primary antibodies (anti-α-smooth muscle actin (α-SMA), 1:100, Abcam; or anti-RelA, 1:100, Cell Signaling Technology, Danvers, MA, USA) overnight at 4 °C, and signals were visualized using DAB substrate (Santa Cruz Biotechnology, Dallas, TX, USA).

For immunofluorescence (IF), sections were incubated overnight at 4 °C with primary antibodies against F4/80 (1:100), Ki67 (1:100), 8-OHdG (1:100), phospho-c-Jun (Ser63, 1:100), α-fetoprotein (AFP, 1:100), Glutamine synthetase (GS, 1:100), phospho-H2AX (Ser139, 1:100), CYP2E1 (1:100), and HNF4α (1:100) (all from Cell Signaling Technology). Fluorescent signals were detected using Cy3- or FITC-conjugated secondary antibodies (Jackson Immuno Research, West Grove, PA, USA), and nuclei were counterstained with DAPI. Images were acquired using a BZ-9000 microscope (KEYENCE, Osaka, Japan).

Quantification was performed using ImageJ (Fiji distribution; version 1.54g). For each mouse, four randomly selected fields were analyzed. Nuclear signal intensities were quantified for Ki67, phospho-c-Jun, phospho-H2AX, and HNF4α, whereas cytoplasmic CYP2E1 signal intensity was quantified per hepatocyte. Imaging and analysis parameters were kept constant across all groups. After background subtraction, mean fluorescence intensity was calculated per nucleus (for nuclear markers) or per hepatocyte (for CYP2E1). For each mouse, 50 nuclei/hepatocytes were quantified to generate a single biological replicate (*n* = 4/group). Quantification procedures for additional markers (e.g., F4/80 and 8-OHdG) are described in the corresponding figure legends. Formal blinding was not performed; quantification was conducted using predefined criteria and fixed imaging/analysis parameters across all groups.

### 2.9. TUNEL Assay

Apoptotic cells were detected using the In Situ Cell Death Detection Kit, Fluorescein (Sigma-Aldrich, St. Louis, MO, USA), according to the manufacturer’s instructions. Fluorescent images were obtained using a BZ-9000 microscope (KEYENCE), and TUNEL-positive nuclei were quantified in four randomly selected fields per section.

### 2.10. Western Blotting

Liver tissues were homogenized in lysis buffer (50 mM Tris-HCl, 1 mM EDTA, 150 mM NaCl, 1% Triton X-100, 1 mM DTT, and protease inhibitor cocktail). After centrifugation, supernatants were subjected to SDS–PAGE and transferred onto PVDF membranes. Membranes were blocked with 3% skim milk in PBST (PBS containing 0.1% Tween 20) for 1 h, then incubated with primary antibodies for 2 h at room temperature, followed by HRP-conjugated secondary antibodies (1:5000; Cell Signaling Technology). Protein bands were detected using SuperSignal West Pico Chemiluminescent Substrate (Thermo Fisher Scientific, Waltham, MA, USA) and visualized on X-ray film (FUJIFILM Wako Pure Chemical Corp.).

Primary antibodies: anti-IKKβ (Abcam, 1:1000), anti-Flag (Sigma-Aldrich, 1:3000), anti-α-Tubulin (1:2000; Cell Signaling Technology), anti-Lamin B1 (1:1000; Cell Signaling Technology), anti-CYP2E1 (Abcam, Cambridge, UK, 1:1000), anti-PGC-1α (Abcam, 1:1000), anti-Glutamine synthetase (GS) (Abcam, 1:1000), and anti-β-actin (Santa Cruz Biotechnology, 1:5000).

### 2.11. RNA Extraction and RT-qPCR

Total RNA was extracted from livers homogenized in TRIzol (Invitrogen, Waltham, MA, USA) and reverse transcribed using the Verso cDNA Synthesis Kit (Thermo Fisher Scientific). The reverse transcription cycle was performed at 42 °C for 40 min, followed by 95 °C for 2 min. RT-qPCR was carried out using the CFX96 Real-Time PCR System (Bio-Rad, Hercules, CA, USA) and SYBR Premix Ex Taq (Takara Bio Inc., Shiga, Japan). PCR amplification consisted of two steps: denaturation at 95 °C for 5 s and annealing/extension at 60 °C for 30 s, repeated for 40 cycles. Relative gene expression was normalized to Cph mRNA levels and calculated using the comparative Ct (ΔΔCt) method. Primer sequences were verified for specificity using NCBI Primer-BLAST and synthesized by FASMAC (Kanagawa, Japan). Amplification efficiency (90–110%) was confirmed by standard curve analysis. Primer sequences are listed in Supplementary Table S2.

### 2.12. Microarray Analysis

Total RNA was extracted from liver tissues using TRIzol Reagent (Invitrogen) and further purified using RNeasy MinElute Spin Columns (Qiagen, Hilden, Germany). Gene-expression profiling was performed with the GeneChip Mouse Genome 430 2.0 Array (Affymetrix, Santa Clara, CA, USA) according to the manufacturer’s instructions.

For this analysis, RNA from one representative 6-week-old male mouse from each of the four genotypes (*n* = 1 per genotype) was used. This microarray experiment was designed as an exploratory reference dataset to identify genotype-associated transcriptional alterations in the setting of spontaneous hepatitis. In addition, microarray analysis was performed on livers from 8-week-old male C57BL/6J mice, collected 4 h after saline, TNFα, or IL-1β administration (*n* = 1 per condition).

Differentially expressed genes between Tg-IKKβ^Δhep^ and IKKβ^F/F^ livers were analyzed for Gene Ontology (GO) term enrichment using the DAVID Bioinformatics Resources. Enrichment significance was assessed using DAVID’s modified Fisher’s exact test, and multiple-testing correction was applied to control the false discovery rate (FDR). Fold enrichment (FE) values were computed by DAVID based on observed versus expected gene counts for each GO term using the specified background gene set. Results were visualized as bar plots of −log10 (FDR) with FE and the number of genes contributing to each term (*n*) indicated.

Candidate differentially expressed genes were subsequently validated by independent quantitative RT-qPCR analyses using additional biological replicates, as described above. Raw and processed microarray data have been deposited in the NCBI Gene Expression Omnibus (GEO) under accession numbers GSE324983 and GSE324985. GSE324983 includes one representative mouse from each of the four genotypes, whereas GSE324985 includes WT liver samples collected 4 h after saline, TNFα, or IL-1β administration.

### 2.13. Electrophoretic Mobility Shift Assay (EMSA)

Double-stranded synthetic probes for EMSA were prepared by heating equimolar amounts of complementary oligonucleotides at 100 °C for 5 min and gradually cooling to room temperature to allow annealing. The resulting double-stranded fragments contained single-stranded 5′ overhangs, which were end-labeled with [α-^32^P]dCTP (3000 Ci/mmol; PerkinElmer, Waltham, MA, USA) using the Klenow fragment of DNA polymerase I (Takara Bio Inc.). Non-labeled probes were used as cold competitors.

Binding reactions were performed in 20 μL containing 100,000 cpm of labeled probe, 10 μg of nuclear extract, 1 μg poly(dI–dC)·poly(dI–dC), and 40 pmol of unlabeled competitor oligonucleotide when indicated. Reaction mixtures were incubated for 30 min at 37 °C in 1× EMSA binding buffer (10 mM Tris-HCl pH 7.5, 50 mM NaCl, 1 mM DTT, 1 mM EDTA, 5% glycerol). Protein–DNA complexes were separated on 7% native polyacrylamide gels in 0.5× TBE buffer at 150 V for 2 h, dried, and visualized by autoradiography. Oligonucleotide sequences used for EMSA are provided in Supplementary Table S3.

### 2.14. Chromatin Immunoprecipitation (ChIP) and ChIP-Seq

ChIP assays were performed using 50 mg of liver tissue from one representative 6-week-old male mouse per group (*n* = 1 biological replicate per group; see the corresponding figure legends) using a ChIP assay kit (Active Motif, Carlsbad, CA, USA) according to the manufacturer’s instructions. Liver tissues were cross-linked with 1% formaldehyde for 10 min at 37 °C with gentle shaking, and the reaction was quenched with 0.125 M glycine. Chromatin was isolated, sonicated, and immunoprecipitated with 2 μg of control IgG (Santa Cruz Biotechnology), anti-RNA polymerase II (Active Motif), or anti-HNF4α (Abcam). Immunoprecipitated DNA was purified by phenol/chloroform/isoamyl alcohol extraction and ethanol precipitation, and enrichment at target promoters was quantified by ChIP-qPCR as described above using the CFX96 Real-Time PCR System (Bio-Rad) and SYBR Premix Ex Taq (Takara Bio Inc.). Non-precipitated genomic DNA (input chromatin) served as a normalization control. Primer sequences targeting the promoter regions of Cyp2e1, Cyp7a1, Cyp8b1, and Car are provided in Supplementary Table S3.

For ChIP-seq, one representative chromatin sample (*n* = 1 per genotype) was used for library preparation with the TruSeq ChIP Sample Prep Kit (Illumina, San Diego, CA, USA), followed by sequencing on a HiSeq2000 platform (Illumina). Raw reads were aligned to the mouse reference genome (mm10) using Bowtie, and peaks were called using the MACS2 (v2.0.10.20120913). This ChIP-seq experiment was designed as an exploratory reference dataset to characterize genotype-associated HNF4α chromatin occupancy patterns in Tg-IKKβ^Δhep^ mouse liver. Raw and processed data will be deposited in the NCBI Gene Expression Omnibus (GEO) (accession number: GSE322777).

### 2.15. Statistical Analysis

No a priori power calculation was performed. Sample sizes were determined based on conventions in similar mouse studies and experimental feasibility, and the exact *n* for each analysis is provided in the corresponding figure legends. Statistical analyses were performed using GraphPad Prism version 10.6.1 (GraphPad Software). The statistical methods used for each dataset are indicated in the corresponding figure legends and/or relevant Methods subsections. For tumor size outcomes in which multiple tumors arose within a single animal, the mouse was treated as the experimental unit and tumor size was summarized per animal (e.g., maximum or mean diameter per mouse) prior to group comparison; tumor-level distributions are shown for descriptive purposes only. Tumor multiplicity per mouse and total tumor burden per mouse were analyzed at the animal level, with each mouse contributing a single summary value. Tumor incidence (defined as the proportion of mice bearing at least one macroscopic tumor) was compared among groups using Fisher’s exact test. Unless otherwise stated, data are presented as mean ± SEM. Comparisons between two groups were analyzed using an unpaired, two-tailed Student’s *t*-test. Comparisons among multiple groups were analyzed by one-way ANOVA followed by Dunnett’s multiple-comparisons test (for comparisons with a designated control group) or Tukey’s multiple-comparisons test (for all pairwise comparisons), as appropriate. Analyses involving two factors (e.g., genotype and treatment or genotype and age) were performed by two-way ANOVA, with Šídák’s multiple-comparisons test for prespecified post hoc comparisons where appropriate. Survival was analyzed by the Kaplan–Meier method and compared using the log-rank (Mantel–Cox) test; hazard ratios with 95% confidence intervals (CIs) were reported where indicated. Assumptions for parametric tests (normality and homogeneity of variance) were assessed using appropriate tests (e.g., Shapiro–Wilk and Brown–Forsythe tests); when these assumptions were not met, appropriate nonparametric tests (e.g., Kruskal–Wallis with Dunn’s multiple-comparisons test) were used. Unless otherwise specified, all tests were two-sided, and *p* < 0.05 was considered statistically significant. For primary outcome measures and selected key comparisons, effect sizes with 95% CIs are reported in the corresponding figure legends. Statistical significance is indicated as follows: * *p* < 0.05, ** *p* < 0.01, *** *p* < 0.001, and **** *p* < 0.0001 (ns, not significant).

## 3. Results

### 3.1. Generation and Phenotypic Characterization of Tg-IKKβ^Δhep^ Mice Exhibiting Spontaneous Chronic Hepatitis and Progressive Fibrosis

**Figure 1:**
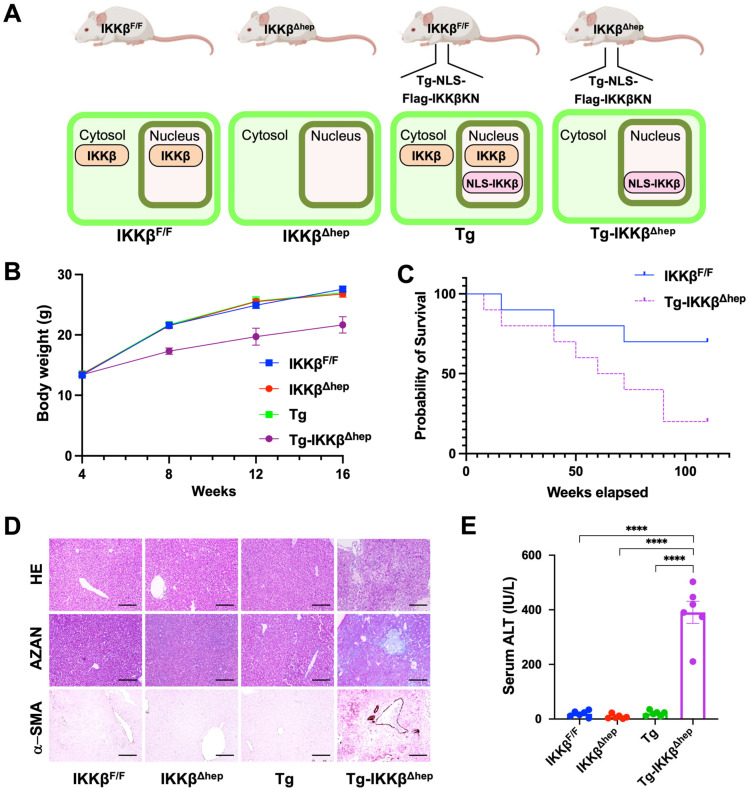
Phenotypic analysis of Tg-IKKβ^Δhep^ mice. (**A**) Schematic overview of the breeding strategy used to generate the four genotypes (IKKβ^F/F^, IKKβ^Δhep^, Tg, and Tg-IKKβ^Δhep^). Created with BioRender.com. (**B**) Body weight from 4 to 16 weeks of age (*n* = 6 per group). (**C**) Kaplan–Meier survival curves for the indicated genotypes (IKKβ^F/F^ and Tg-IKKβ^Δhep^; *n* = 10 per group). (**D**) Representative liver sections stained from 4-week-old mice with hematoxylin and eosin (H&E) and Azan, and immunostained for α-smooth muscle actin (α-SMA). Scale bar: 100 μm. (**E**) Serum alanine aminotransferase (ALT) activity in 4-week-old mice (*n* = 6 per group). Quantitative data (**B**,**E**) are presented as mean ± SEM. For panel (**B**), body weight measurements at each age were obtained from independent cohorts and were analyzed by two-way ANOVA (genotype × age). Survival curves in panel (**C**) were compared using the log-rank (Mantel–Cox) test. The hazard ratio (Tg-IKKβ^Δhep^ vs. IKKβ^F/F^; Mantel–Haenszel) was 3.566 (95% CI, 1.041 to 12.22). Serum ALT activity in panel E was analyzed by one-way ANOVA followed by Dunnett’s multiple-comparisons test using Tg-IKKβ^Δhep^ as the control group. Effect sizes for panel (**E**) are reported as mean differences (Tg-IKKβ^Δhep^−comparator) with 95% confidence intervals (CIs): vs. IKKβ^F/F^, 371.5 U/L (95% CI, 297.6 to 445.3); vs. IKKβ^Δhep^, 381.9 U/L (95% CI, 308.1 to 455.7); vs. Tg, 366.6 U/L (95% CI, 292.8 to 440.4). **** *p* < 0.0001.

To investigate the hepatocyte-intrinsic function of nuclear IKKβ in vivo, we generated transgenic mice expressing a nuclear localization signal-fused, kinase-inactive IKKβ mutant (FLAG–NLS–IKKβ(K44A); NLS-IKKβKN) under the control of the mouse Ikbkb 5′ regulatory region and crossed them with hepatocyte-specific IKKβ-deficient mice to generate Tg-IKKβ^Δhep^ mice (Figure 1A, Supplementary Figure S1A,B) [27]. Immunoblotting confirmed loss of endogenous IKKβ and liver-specific expression of the NLS-IKKβKN transgene (Supplementary Figure S1C,D).

Although Tg-IKKβ^Δhep^ mice were obtained at the expected Mendelian frequency, they exhibited pronounced postnatal growth retardation compared with control groups (Figure 1B and Supplementary Figure S1D). Kaplan–Meier survival analysis demonstrated markedly reduced survival, with approximately 50% of Tg-IKKβ^Δhep^ mice dying within 50 weeks after birth (Figure 1C).

Histopathological analyses revealed spontaneous hepatitis as early as 4 weeks of age, followed by progressive fibrosis by 16 weeks (Figure 1D and Supplementary Figure S2A,B). Azan staining and α-smooth muscle actin (α-SMA) immunohistochemistry demonstrated extensive collagen deposition and hepatic stellate cell activation in Tg-IKKβ^Δhep^ livers (Figure 1D). Immunofluorescence analyses showed increased numbers of cells positive for RelA, TUNEL, F4/80, Ki67, 8-OHdG, and phospho-c-Jun, indicating enhanced hepatocyte proliferation, macrophage accumulation, NF-κB activation, oxidative DNA damage, and apoptosis (Supplementary Figure S3A,B).

Consistent with these histological changes, serum ALT levels were significantly elevated in Tg-IKKβ^Δhep^ mice (Figure 1E and Supplementary Figure S2C). RT-qPCR analyses revealed strong induction of fibrosis- and inflammation-associated genes, including Collagen 1a1, α-Sma, Timp1, Tnfα, and Il6 (Supplementary Figure S4). These in vivo phenotypes are consistent with our previous cell-based findings that nuclear IKKβ promotes stress-induced IκBα ubiquitination and degradation, thereby activating NF-κB while also contributing to cell-death responses [27], and they support the physiological relevance of altered nuclear IKKβ signaling in liver pathophysiology.

### 3.2. DEN-Induced Hepatocarcinogenesis Is Suppressed in Tg-IKKβ^Δhep^ Mice

**Figure 2:**
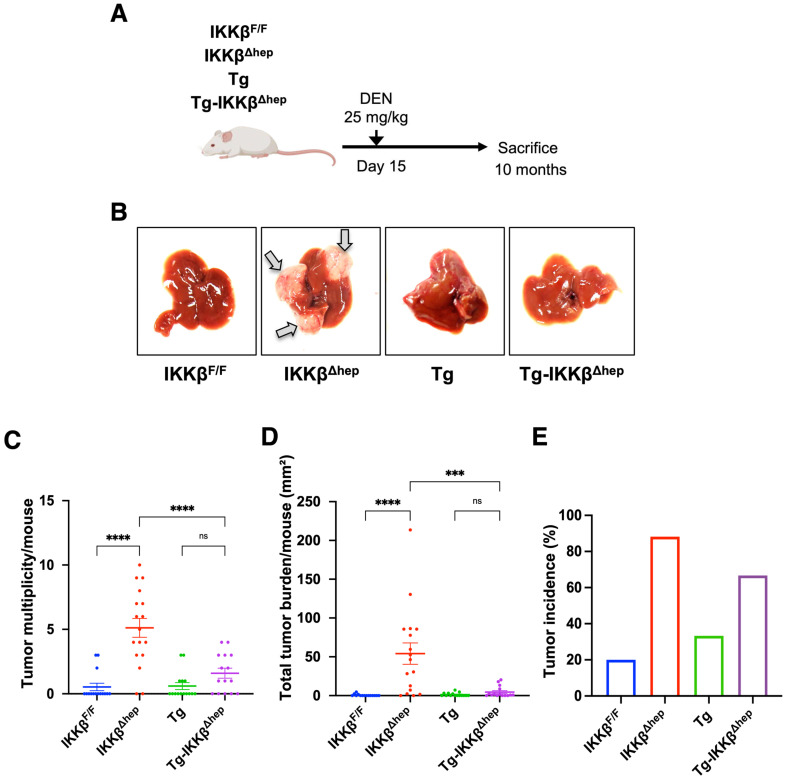
DEN-induced hepatocarcinogenesis in Tg-IKKβ^Δhep^ mice. (**A**) Experimental design for DEN-induced hepatocarcinogenesis: tumor formation was evaluated 10 months after a single intraperitoneal DEN injection (25 mg/kg) administered at postnatal day 15 (P15). (**B**) Representative gross liver images showing DEN-induced liver tumors across the four genotypes (arrows indicate macroscopic tumors). (**C**–**E**) Analyses in 10-month-old male mice: IKKβ^F/F^ (*n* = 15), IKKβ^Δhep^ (*n* = 17), Tg (*n* = 15), and Tg-IKKβ^Δhep^ (*n* = 15). (**C**) Tumor multiplicity per mouse. (**D**) Total tumor burden per mouse (mm^2^), calculated as the sum of tumor areas Σπ(d/2)^2^ for each mouse, with mice without tumors assigned a value of 0. (**E**) Tumor incidence (tumor-bearing/total mice), defined as the proportion of mice bearing ≥1 macroscopic tumor. Tumor multiplicity and total tumor burden are presented as mean ± SEM. Statistical significance for panels (**C**,**D**) was assessed by one-way ANOVA followed by the appropriate multiple-comparisons test, as described in Section 2.15. Tumor incidence in panel (**E**) was compared across the four groups by Fisher’s exact test and differed significantly among groups (*p* < 0.0001). *** *p* < 0.001, **** *p* < 0.0001; ns, not significant.

To assess susceptibility to chemical hepatocarcinogenesis, DEN-induced tumor formation was compared among four genotypes (IKKβ^F/F^, IKKβ^Δhep^, Tg, and Tg-IKKβ^Δhep^) (Figure 2A). Consistent with previous reports, hepatocyte-specific IKKβ deletion (IKKβ^Δhep^) markedly increased tumor multiplicity per mouse and total tumor burden per mouse (Figure 2C,D) [16]. In contrast, Tg-IKKβ^Δhep^ mice exhibited lower tumor multiplicity per mouse and lower total tumor burden per mouse than IKKβ^Δhep^ mice, indicating that DEN-induced hepatocarcinogenesis was attenuated in Tg-IKKβ^Δhep^ livers (Figure 2B–D). Tumor incidence (tumor-bearing/total mice) was also evaluated to complement per-mouse analyses of tumor multiplicity and total tumor burden and to account for animals without macroscopic tumors (Figure 2E).

Collectively, these findings demonstrate that DEN-induced hepatocarcinogenesis is attenuated in Tg-IKKβ^Δhep^ mice despite the presence of chronic inflammation and fibrosis. We therefore next examined whether this phenotype was associated with reduced acute DEN-induced liver injury and diminished DNA damage responses.

### 3.3. Reduced DEN-Induced Liver Injury and DNA Damage in Tg-IKKβ^Δhep^ Mice Is Associated with Diminished Pericentral CYP2E1 Expression

**Figure 3:**
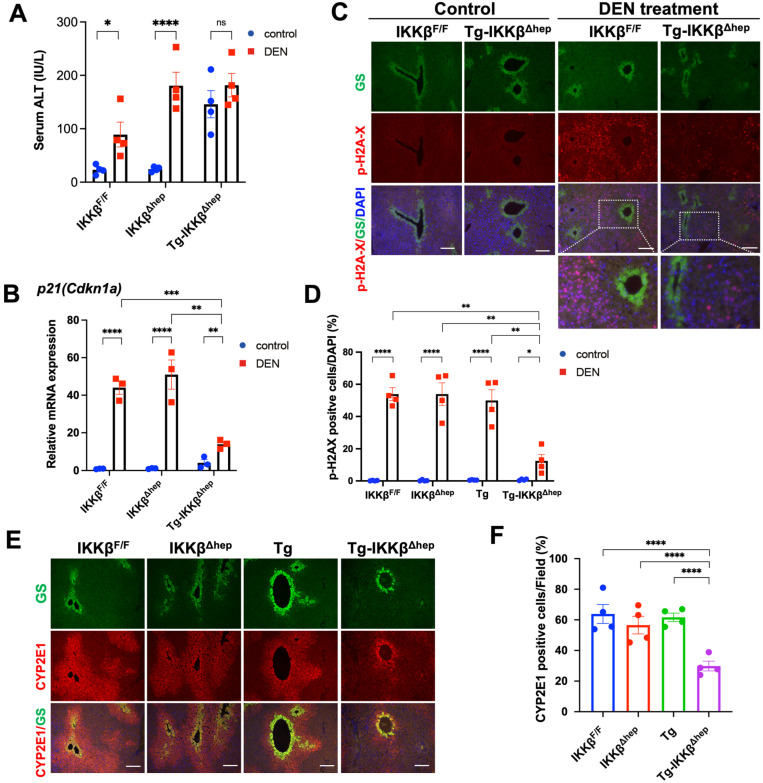
Attenuated DEN-induced DNA damage in Tg-IKKβ^Δhep^ mice. (**A**) Serum ALT activity at 48 h after DEN administration (50 mg/kg, i.p.) in 8-week-old mice (*n* = 4 per group). (**B**) Hepatic p21 (Cdkn1a) mRNA levels at 48 h after DEN (RT-qPCR; *n* = 3 per group). (**C**) Representative immunostaining for phosphorylated H2AX (Ser139) at 48 h after DEN, with enlarged views of the pericentral region defined by glutamine synthetase (GS)-positive hepatocytes. Scale bar: 100 μm. (**D**) Quantification of p-H2AX-positive cells. (**E**) CYP2E1 immunostaining in livers from the four genotypes, with enlarged pericentral views. (**F**) Quantification of CYP2E1-positive area fraction within the GS-defined pericentral compartment. Data are presented as mean ± SEM. * *p* < 0.05, ** *p* < 0.01, *** *p* < 0.001, **** *p* < 0.0001; ns, not significant.

To assess acute DEN-induced liver injury, serum ALT levels were measured 48 h after DEN administration (Figure 3A). ALT levels increased in IKKβ^F/F^ and Tg mice and were highest in IKKβ^Δhep^ mice. In contrast, Tg-IKKβ^Δhep^ mice showed only minimal ALT elevation, indicating marked attenuation of DEN-induced hepatocyte injury. Because DEN-induced genotoxic stress activates the p53 pathway and induces p53 target genes such as p21 [13,14,15], we next examined p21 induction after DEN exposure. Although p21 and p-H2AX provide indirect readouts rather than direct measurements of DEN-derived DNA adducts, p21 induction was strongly attenuated in Tg-IKKβ^Δhep^ mice (Figure 3B).

Because DEN is predominantly bioactivated in the pericentral (zone 3) region and induces DNA damage in this compartment [13,14,15], pericentral DNA damage was evaluated by phosphorylated H2AX (p-H2AX; γH2AX) staining (Figure 3C,D and Supplementary Figure S5A). DEN induced robust pericentral p-H2AX signals in control livers, whereas p-H2AX positivity was markedly reduced in Tg-IKKβ^Δhep^ livers. Notably, γH2AX staining and p21 induction provide indirect surrogate readouts of DEN-induced genotoxic stress rather than direct measurements of DEN-derived DNA adduct formation.

The pericentral (zone 3) compartment was defined by glutamine synthetase (GS), a canonical zone 3 marker, and analyses were performed with explicit attention to zonal architecture. Quantitative immunohistochemical analysis of GS showed that, in Tg-IKKβ^Δhep^ livers, the overall mean gray value measured across fixed-size ROIs centered on central veins was not significantly altered, whereas GS-positive area (%) was significantly reduced, suggesting a spatial contraction of the GS-positive pericentral compartment (Supplementary Figure S5B). Importantly, even when the analysis was restricted to the GS-positive area, pericentral CYP2E1 protein was still markedly reduced in Tg-IKKβ^Δhep^ livers (Figure 3E,F). This finding was further supported by immunoblotting, which showed decreased hepatic CYP2E1 protein abundance (Supplementary Figure S6). Taken together, these results indicate that reduced pericentral CYP2E1 expression in Tg-IKKβ^Δhep^ mice is consistent with diminished DEN bioactivation and could contribute to attenuated hepatocyte injury and downstream DNA damage responses.

### 3.4. Broad Suppression of Hepatic Cyp Genes and Reduced HNF4α/Pol II Promoter Occupancy in Tg-IKKβ^Δhep^ Liver

To define the molecular basis of resistance to DEN-induced hepatocarcinogenesis, we first performed global transcriptomic profiling. Notably, this transcriptome analysis was used as an exploratory screen, and key findings were subsequently validated by RT-qPCR and ChIP as described below. Heatmap visualization revealed coordinated suppression of zone 3 xenobiotic- and bile acid-related CYP programs, together with broad repression of metabolic pathways, including mitochondrial respiration/oxidative phosphorylation (OXPHOS) gene sets, in Tg-IKKβ^Δhep^ livers (Supplementary Figure S7A,B). In contrast, signatures of inflammation and extracellular matrix (ECM) remodeling were enriched (Supplementary Figure S7D), consistent with the coexistence of metabolic repression and a pro-inflammatory tissue state. Gene Ontology (GO) enrichment analysis further supported this dichotomy: pathways related to inflammation and tissue remodeling were overrepresented among upregulated genes, whereas metabolic and redox-related processes were enriched among downregulated genes (Supplementary Figure S8).

**Figure 4:**
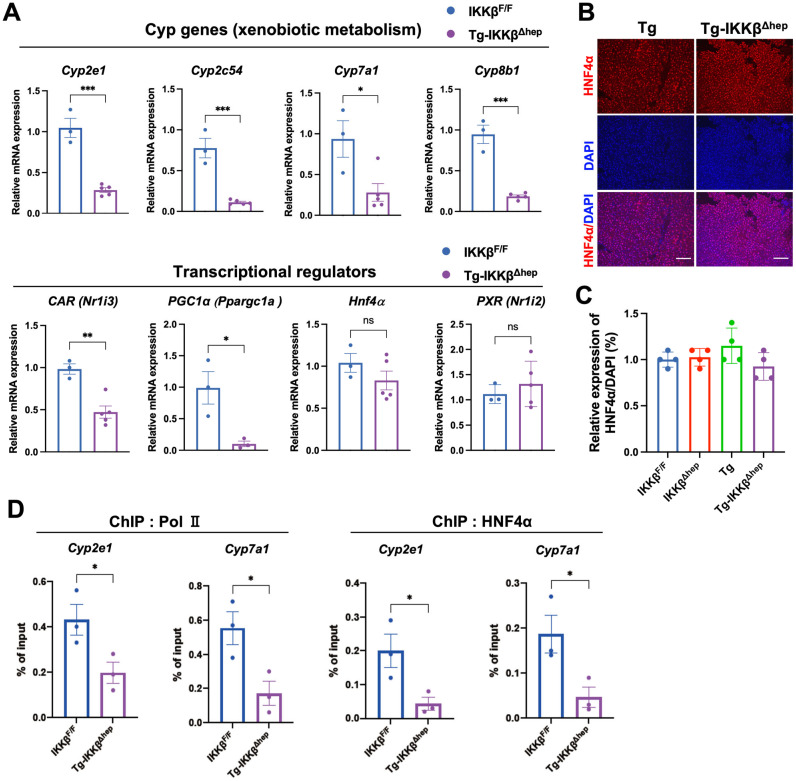
Reduced pericentral CYP2E1 expression and impaired HNF4α transcriptional activity in Tg-IKKβ^Δhep^ livers. (**A**) RT-qPCR analysis of Cyp genes and nuclear receptor-related genes in livers from 6-week-old mice of IKKβ^F/F^ mice (*n* = 3) and Tg-IKKβ^Δhep^ mice (*n* = 5), normalized to Cph. (**B**) Representative immunofluorescence staining for HNF4α. Scale bar: 100 μm. (**C**) Quantification of HNF4α-positive hepatocytes (*n* = 4 per group). (**D**) ChIP-qPCR analysis of RNA polymerase II (Pol II) and HNF4α occupancy at the indicated promoters (including the Cyp2e1 and Cyp7a1 promoters) in livers from 6-week-old mice. Primer information is provided in Supplementary Table S3. ChIP was performed using chromatin from IKKβ^F/F^ and Tg-IKKβ^Δhep^ livers (*n* = 3 per group). Data are presented as mean ± SEM for quantitative analyses unless otherwise stated. Panels (**A**,**C**,**D**) were analyzed using an unpaired, two-tailed Student’s t-test. Representative images in panel (**B**) are shown for illustration. * *p* < 0.05, ** *p* < 0.01, *** *p* < 0.001; ns, not significant.

Based on these global analyses, we next validated expression of upstream regulators and xenobiotic metabolism-related genes by RT-qPCR. Multiple cytochrome P450 (Cyp) genes involved in xenobiotic and bile acid metabolism (Cyp2e1, Cyp2c54, Cyp7a1, and Cyp8b1) were significantly downregulated in Tg-IKKβ^Δhep^ livers compared with IKKβ^F/F^ controls (Figure 4A). In the same samples, CAR (Nr1i3) and the metabolic coactivator PGC-1α (Ppargc1a) were also significantly reduced, whereas Hnf4a and PXR (Nr1i2) mRNA levels showed no marked changes (Figure 4A and Supplementary Figure S7C).

Because HNF4α is a central regulator of hepatic metabolic gene expression, we assessed HNF4α protein expression by immunofluorescence. Representative images showed no obvious loss of nuclear HNF4α signal in Tg-IKKβ^Δhep^ livers (Figure 4B,C and Supplementary Figure S9A). However, because HNF4α abundance does not necessarily reflect its transcriptional activity, we examined its DNA-binding capacity. EMSA indicated a trend toward reduced HNF4α DNA-binding activity in Tg-IKKβ^Δhep^ livers, suggesting impaired transcriptional output not explained solely by changes in HNF4α abundance (Supplementary Figure S9B). We therefore examined promoter-level transcriptional engagement. Chromatin immunoprecipitation (ChIP) assays demonstrated reduced occupancy of both HNF4α and RNA polymerase II (Pol II) at the Cyp2e1 and Cyp7a1 promoters in Tg-IKKβ^Δhep^ livers (Figure 4D). Additional promoter analyses for Cyp8b1 and Car are provided in Supplementary Figure S9C.

Collectively, these data indicate that, despite a persistent inflammatory tissue state, Tg-IKKβ^Δhep^ livers exhibit broad repression of xenobiotic metabolism programs together with reduced HNF4α/Pol II promoter engagement, providing a mechanistic basis for diminished Cyp-dependent carcinogen metabolism.

### 3.5. Acute TNFα/IL-1β Stimulation Suppresses Hepatic Cyp Genes via Reduced PGC-1α Expression and Pol II Promoter Occupancy

**Figure 5:**
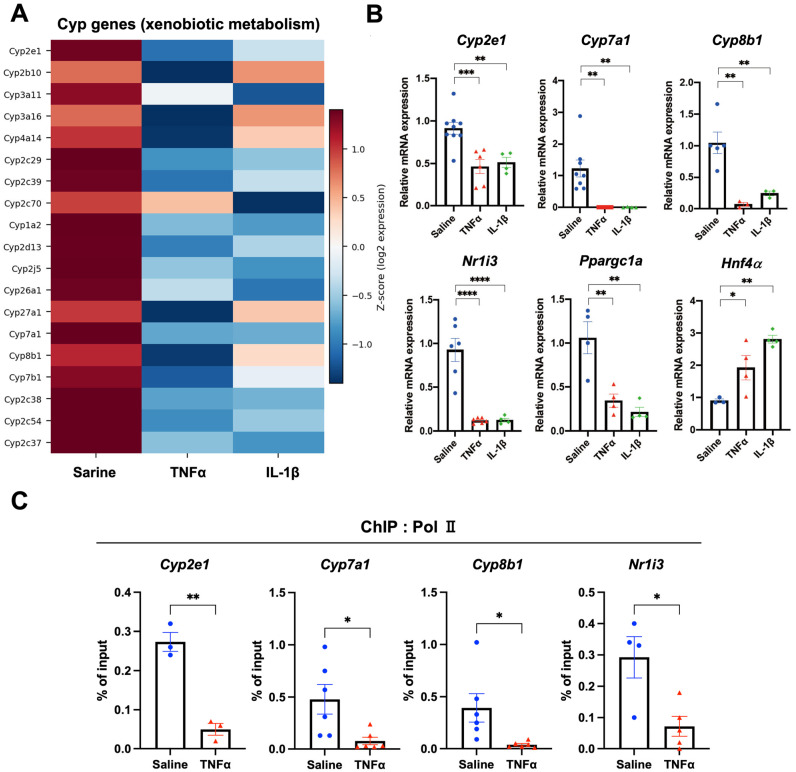
Acute inflammatory cytokines suppress hepatic drug-metabolism genes and Pol II recruitment. (**A**) Heatmap showing changes in xenobiotic/bile acid metabolism-related Cyp genes (including Cyp2e1, Cyp7a1, and Cyp8b1) at 4 h after TNFα or IL-1β administration, based on an exploratory microarray analysis. Gene-level results, including downregulation of nuclear receptor pathways (e.g., CAR (Nr1i3)), mitochondrial respiration/OXPHOS genes, and metabolic regulators (e.g., PGC-1α (Ppargc1a)), as well as induction of acute inflammatory markers (e.g., S100a8/S100a9), are provided in Supplementary Figure S10. (**B**) RT-qPCR analysis of Cyp genes and nuclear receptor genes in livers harvested 4 h after TNFα or IL-1β injection in 8-week-old WT mice: saline (*n* = 8), TNFα (*n* = 6), and IL-1β (*n* = 4) (normalized to Cph). (**C**) ChIP-qPCR analysis of Pol II promoter occupancy (Cyp2e1, Cyp7a1, Cyp8b1, CAR (Nr1i3) and Ppara) under the same conditions: saline (*n* = 3–6), TNFα (*n* = 3–6), and IL-1β (*n* = 3–5). Data are presented as mean ± SEM unless otherwise stated. Panels B and C were analyzed by one-way ANOVA followed by Dunnett’s multiple-comparisons test (vs. saline), as appropriate; unpaired, two-tailed Student’s *t*-test was used for two-group comparisons where applicable. Panel A shows exploratory microarray results. * *p* < 0.05, ** *p* < 0.01, *** *p* < 0.001, **** *p* < 0.0001.

To test whether acute inflammatory signals can recapitulate the transcriptional state observed in Tg-IKKβ^Δhep^ livers, we analyzed mouse livers after administration of TNFα or IL-1β. Global microarray profiling revealed broad transcriptional remodeling characterized by robust induction of inflammatory gene programs and concomitant repression of metabolic genes, including marked suppression of drug metabolism-associated Cyp gene expression (Figure 5A). Notably, the microarray analysis was exploratory, and key findings were validated by RT-qPCR and ChIP as described below. Heatmap visualization of the top differentially expressed genes showed strong upregulation of acute inflammatory markers (e.g., S100a8/S100a9, Lcn2, and Saa family members) following both cytokine stimuli, whereas multiple hepatic metabolic regulators and xenobiotic metabolism genes were downregulated (Figure 5A and Supplementary Figure S10).

RT-qPCR confirmed downregulation of Cyp2a1, Cyp7a1, Cyp8b1, and Car (Figure 5B). In the same samples, Hnf4a mRNA levels were unchanged or modestly increased, whereas PGC-1α (Ppargc1a), a major coactivator of HNF4α, was significantly reduced (Figure 5B). Furthermore, ChIP assays demonstrated that Pol II occupancy at promoters of Cyp2e1, Cyp7a1, Cyp8b1, and Car was strongly decreased after TNFα stimulation (Figure 5C). These results indicate that acute inflammatory stimulation can induce coordinated suppression of xenobiotic metabolism gene programs together with promoter-proximal transcriptional repression. In line with this, genome browser visualization of Pol II ChIP-seq tracks supported this promoter-proximal repression. Because the absolute Pol II ChIP-seq signal at the Cyp7a1 and Cyp8b1 loci was relatively low, locus-specific changes were primarily supported by Pol II ChIP-qPCR quantification (Figure 5C). At representative loci including Cyp2e1 (and low-signal loci such as Cyp7a1 and Cyp8b1), genome browser tracks were consistent with reduced Pol II occupancy in Tg-IKKβ^Δhep^ livers and in WT livers after TNFα administration (Supplementary Figure S11). Together with the ChIP-qPCR data, these observations strongly support the conclusion that inflammatory signaling, either acutely induced by TNFα or chronically present in Tg-IKKβ^Δhep^ liver, attenuates Pol II recruitment/engagement at key xenobiotic and bile acid metabolism genes, thereby contributing to broad suppression of hepatocyte detoxification programs.

### 3.6. Chronic CCl_4_ Injury Downregulates Pericentral CYP2E1 and Suppresses DEN-Induced Hepatocarcinogenesis

**Figure 6:**
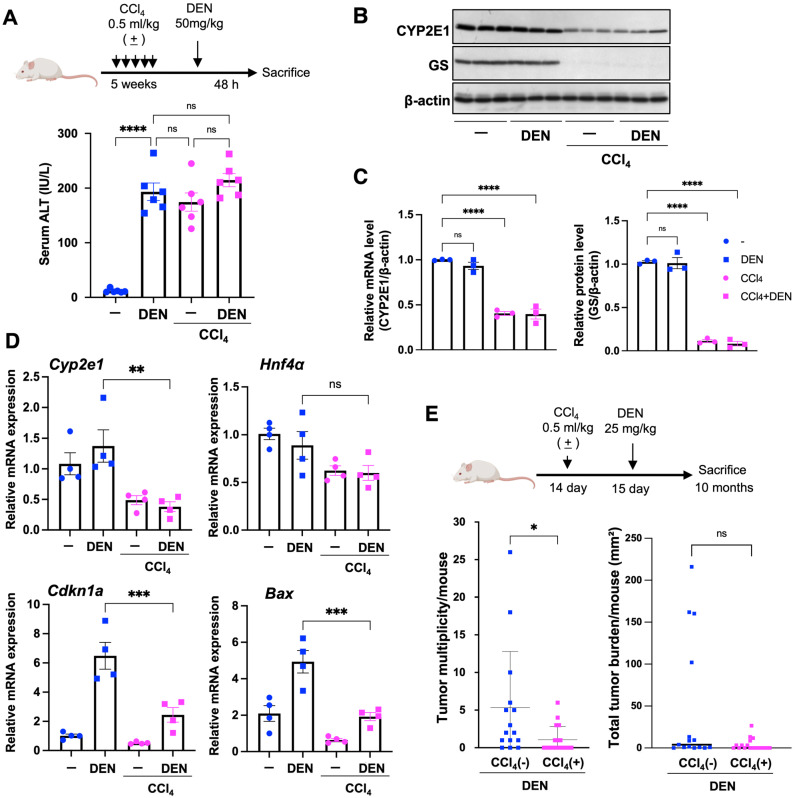
Chronic CCl_4_ reduces hepatic CYP2E1/GS and alters DEN-related outcomes. (**A**) Experimental scheme (top) and serum ALT activity (bottom) across four groups (untreated, DEN alone, CCl_4_ alone, and DEN after chronic CCl_4_) following chronic CCl_4_ treatment (i.p., twice per week for 5 weeks) (*n* = 6 per group). (**B**) Immunoblot analysis of CYP2E1 and glutamine synthetase (GS) in liver extracts; β-actin was used as a loading control. (**C**) Densitometric quantification of CYP2E1 and GS normalized to β-actin (*n* = 3 per group). (**D**) RT-qPCR analysis of Cyp2e1, Hnf4a, Cdkn1a (p21), and Bax, normalized to Cph and shown relative to the baseline defined within each panel. (**E**) Experimental scheme (top) and tumor multiplicity per mouse and total tumor burden per mouse (mm^2^) in 10-month-old mice after DEN administration: DEN alone [CCl_4_(-)] (*n* = 15) and DEN after chronic CCl_4_ [CCl_4_(+)] (*n* = 18). Tumor multiplicity per mouse was defined as the number of tumors per mouse. Total tumor burden per mouse was calculated as the sum of tumor areas Σπ(d/2)^2^ for each mouse; mice without tumors were assigned a value of 0. Data are presented as mean ± SEM unless otherwise stated. Statistical significance was assessed as described in Section 2.15. * *p* < 0.05, ** *p* < 0.01, *** *p* < 0.001, **** *p* < 0.0001; ns, not significant.

To examine how chronic liver injury affects pericentral (zone 3) xenobiotic-metabolizing capacity, we assigned mice to four groups (untreated, DEN alone, CCl_4_ alone, and CCl_4_ + DEN) and applied a well-established chronic CCl_4_ protocol (intraperitoneal injections, twice per week for 5 weeks) [32]. Serum ALT levels were significantly elevated in the CCl_4_-treated groups (CCl_4_ alone and CCl_4_ + DEN) compared with untreated controls, confirming establishment of a chronic hepatitis model (Figure 6A). We then measured serum ALT 48 h after DEN administration and found that the ALT elevation observed in the DEN-alone group was attenuated in the CCl_4_ + DEN group (Figure 6A).

Immunoblot analysis of the same liver tissues showed that CYP2E1 protein expression was significantly reduced in the CCl_4_ alone and CCl_4_ + DEN groups (Figure 6B), which was corroborated by densitometric quantification (Figure 6C). Glutamine synthetase (GS) was also markedly decreased (Figure 6C). In agreement, RT-qPCR confirmed reduced Cyp2e1 mRNA levels in the CCl_4_ alone and CCl_4_ + DEN groups, whereas Hnf4a mRNA levels were not significantly altered (Figure 6D). In addition, analysis of p53 target genes after DEN administration showed that induction of p21 and Bax observed in the DEN-alone group was suppressed in the CCl_4_ + DEN group (Figure 6D).

Finally, tumor formation was evaluated 10 months after DEN administration. Mice pretreated with CCl_4_ (CCl_4_ + DEN) showed reduced tumor multiplicity per mouse, whereas total tumor burden per mouse tended to be lower but did not reach statistical significance (Figure 6E). Collectively, these findings indicate that chronic CCl_4_-induced liver injury is associated with suppression of pericentral metabolic programs and attenuation of DEN-related genotoxic and tumorigenic outcomes.

## 4. Discussion

In this study, perturbation of hepatocyte IKKβ signaling caused spontaneous chronic hepatitis and progressive fibrosis yet paradoxically suppressed DEN-induced hepatocarcinogenesis. Despite a strongly inflammatory hepatic milieu characterized by hepatocyte death, compensatory proliferation, and oxidative stress, Tg-IKKβ^Δhep^ mice showed markedly reduced pericentral capacity for procarcinogen activation. Consistent with this, DEN-triggered liver injury, DNA-damage responses, tumor multiplicity per mouse, and total tumor burden per mouse were all reduced. These findings argue against a simple model in which inflammation uniformly promotes cancer and instead support a context-dependent mechanism in which chronic inflammatory injury suppresses hepatic metabolic programs that are required for chemical hepatocarcinogenesis.

### 4.1. Context-Dependent Roles of IKKβ Signaling in Hepatocarcinogenesis

The IKKβ/NF-κB pathway integrates inflammatory and survival signaling in liver disease and HCC, but its consequences depend on etiology, cell type, and disease stage [20,21,22,23,24,25,26]. Hepatocyte-specific loss of IKKβ can enhance ROS accumulation, hepatocyte death, and cytokine-driven compensatory proliferation, thereby promoting DEN-induced hepatocarcinogenesis [16,25]. Our findings add another layer of context dependence: aberrant IKKβ signaling can exacerbate chronic liver injury while simultaneously reducing the metabolic competence required for procarcinogen activation at the initiation stage.

The Tg-IKKβ^Δhep^ model was developed to test whether nuclear IKKβ, previously implicated in stress-induced NF-κB activation and cell-death responses in cultured cells, also functions similarly in hepatocytes in vivo [27]. In these mice, NF-κB activation and hepatocyte death were accompanied by pronounced hepatitis and fibrosis, together with macrophage infiltration and oxidative DNA damage. Yet DEN-induced HCC was suppressed despite this strong inflammatory and regenerative pressure, indicating that inflammatory intensity alone does not determine the outcome of chemical hepatocarcinogenesis. Rather, metabolic competence in the pericentral compartment, where DEN is bioactivated, appears to be a key determinant of initiation susceptibility.

### 4.2. Attenuated DEN-Induced DNA Damage Responses and Impaired Cyp–HNF4α Axis Function

DEN is metabolically activated by CYP2E1 in pericentral (zone 3) hepatocytes, generating DNA-reactive intermediates that induce tissue injury and DNA damage [13,14,15,33]. In Tg-IKKβ^Δhep^ livers, pericentral CYP2E1 was markedly reduced, and DEN-induced ALT elevation, p-H2AX induction, and activation of p53 target genes such as p21 were all blunted. Because these endpoints are indirect readouts rather than direct measurements of DEN-derived DNA adducts, they do not by themselves prove reduced bioactivation. However, they are fully consistent with a model in which insufficient genotoxic input constrains tumor initiation even in an inflammatory microenvironment.

This repression was not limited to Cyp2e1 but extended to multiple Cyp genes, including Cyp7a1 and Cyp8b1, suggesting coordinated suppression of hepatic xenobiotic-metabolism and nuclear-receptor programs rather than an isolated change in a single enzyme. HNF4α is a major regulator of hepatic CYP expression [34,35,36,37,38,39,40,41,42,43,44], and in our model Hnf4a mRNA and total HNF4α protein abundance were largely preserved, whereas EMSA suggested reduced DNA binding and ChIP showed decreased HNF4α and RNA polymerase II occupancy at Cyp and Car loci. These data therefore support reduced functional output of HNF4α, reflected by weaker promoter engagement and Pol II recruitment, rather than simple quantitative loss of HNF4α itself. This interpretation is also consistent with prior evidence that HNF4α-dependent transactivation can be functionally antagonized by AP-1 [45].

### 4.3. Inflammation-Induced Metabolic Suppression as a Brake on Early Chemical Hepatocarcinogenesis

GS-positive area (%) was reduced in Tg-IKKβ^Δhep^ livers, suggesting contraction of the GS-positive pericentral compartment. Importantly, however, CYP2E1 reduction persisted even when quantified within the GS-defined pericentral area, indicating that the phenotype cannot be explained solely by zonation remodeling and likely also reflects suppression of CYP2E1 within the residual pericentral compartment. The paradoxical phenotype observed here is therefore best explained by inflammation-associated metabolic remodeling. Chronic liver injury is known to perturb hepatic zonation and downregulate pericentral metabolic genes such as Cyp2e1 and GS [7,8,9,10,11,12,46,47,48,49,50]. In Tg-IKKβ^Δhep^ mice, Cyp2e1 reduction was most pronounced in the pericentral region, and a similar pattern was reproduced in an independent chronic CCl_4_ injury model.

Together, these findings suggest that chronic inflammation can exert opposing effects on chemical hepatocarcinogenesis: it may enhance tumor-promoting processes during promotion while suppressing tumor initiation by reducing in vivo procarcinogen activation. This interpretation extends the established inflammation–compensatory proliferation–cancer framework [16,26] by placing metabolic activation upstream of DNA damage as an additional rate-limiting determinant of initiation. Consistent with this model, acute TNFα/IL-1β stimulation reproduced downregulation of Cyp/Car genes, suppression of PGC-1α, and reduced Pol II occupancy at target promoters, in line with prior evidence that inflammatory cytokines can antagonize CAR-dependent hepatic detoxification programs [51]. In addition, reciprocal crosstalk between NF-κB and xenobiotic nuclear receptor signaling has also been reported, and NF-κB activation can suppress PXR-dependent transcriptional programs under inflammatory conditions [52]. The chronic CCl_4_ model supported the same overall interpretation: tumor multiplicity per mouse decreased significantly, whereas total tumor burden per mouse showed only a downward trend, suggesting that inflammatory remodeling can measurably restrain initiation even when its effect on overall burden is more modest in an independent cohort.

### 4.4. p53 Signaling and Metabolic Reprogramming in a Hierarchical Model of Tumor Initiation

For DNA-damaged hepatocytes to persist as hepatocyte progenitor-like cells (HcPCs), evasion of p53-dependent apoptosis or senescence is required, and macrophage-derived growth signals can attenuate p53 responses through a CD44-dependent pathway [19]. In our model, DEN-induced expression of p53 target genes (p21, Bax) was reduced in Tg-IKKβ^Δhep^ livers and after chronic CCl_4_ injury. The simpler interpretation, however, is reduced upstream DNA-damage input rather than active suppression of the p53 pathway itself. In this sense, our data do not contradict the p53-evasion model; rather, they place an inflammation-associated metabolic gate upstream of p53 checkpoint activation.

More broadly, the observed Cyp2e1 reduction and impaired HNF4α transcriptional output support the principle that metabolic state can modulate the probability of malignant transformation across liver disease contexts [53,54,55].

### 4.5. IKKβ Signaling as a Link Between Inflammation, Metabolism, and Genome Stability

In summary, perturbation of hepatocyte IKKβ signaling can intensify inflammatory injury and regenerative pressure while simultaneously reducing pericentral xenobiotic-metabolism programs and potentially limiting genotoxic input during initiation. This phenotype cannot be explained by the simple assumption that more inflammation necessarily yields more tumor promotion; zone-specific metabolic state is also a major determinant of susceptibility to chemical carcinogenesis.

These findings help reconcile apparently conflicting reports in the IKKβ/NF-κB literature by highlighting carcinogenesis stage (initiation versus promotion) and metabolic context (capacity for procarcinogen activation) as key variables. Depending on cell type, disease setting, and timing, IKKβ activity may either promote tumor formation through inflammation and compensatory proliferation or, as observed here under severe chronic inflammation, may be associated with reduced pericentral metabolic competence and lower genotoxic input at initiation.

The respective strengths and limitations of genetic versus pharmacological IKKβ suppression also merit consideration. Genetic models provide cell-type specificity and stable long-term pathway modulation that are useful for mechanistic dissection, but lifelong perturbation can induce compensatory rewiring and does not directly mimic therapeutic intervention. By contrast, pharmacological inhibition offers temporal control and greater translational relevance, yet systemic IKKβ-NF-κB inhibition remains challenging because NF-κB is essential for host defense and tissue homeostasis, leaving a narrow therapeutic window [56].

From a translational perspective, CYP2E1-dependent carcinogen bioactivation is shaped by common exposures, including chronic alcohol use and metabolic liver disease [57,58]. Lifestyle measures that limit CYP2E1 induction could therefore reduce CYP2E1-driven genotoxic input. Pharmacological attenuation of CYP2E1 is also conceivable—for example, chlormethiazole-mediated inhibition in an alcohol-fed rat DEN model [33]—but any CYP2E1-targeted strategy would require careful evaluation of safety, drug–drug interactions, and context-specific effects.

### 4.6. Limitations and Future Directions

While our data support an inflammation-driven “metabolic gatekeeping” framework, they do not yet establish causality, and several limitations should be addressed in future studies. First, we did not directly quantify DEN-derived DNA adducts (e.g., O6-ethylguanine), DEN metabolites, or CYP2E1 enzymatic activity in liver microsomes. Future work should quantify alkylated DNA adducts by LC–MS/MS at early time points after DEN exposure and assess CYP2E1-dependent microsomal activity to directly validate differences in genotoxic input [14,15,59,60].

Second, our genome-wide microarray and ChIP-seq datasets were generated from one representative mouse per genotype as exploratory reference profiles. Accordingly, pathway-level repression and chromatin-occupancy patterns should be interpreted cautiously, and we rely primarily on independent validation experiments (RT-qPCR and locus-specific ChIP-qPCR) and phenotypic readouts.

Third, chronic inflammation and fibrosis can remodel lobular architecture and metabolic zonation. Thus, reduced pericentral metabolic programs such as Cyp2e1 and GS may reflect not only cytokine-driven transcriptional repression but also injury-associated remodeling of zonal identity [7,8,9,10,11,12,46,47,48,49,50]. In our histological analyses, central veins remained readily identifiable in Tg-IKKβ^Δhep^ livers, arguing against global collapse of lobular landmarks. However, GS fluorescence was heterogeneous and attenuated in subsets of pericentral hepatocytes, consistent with reduced GS protein abundance by immunoblotting. The modest downward trend in GS-positive pericentral area should therefore be interpreted cautiously, as it may reflect weaker marker intensity in injured hepatocytes rather than complete loss of the pericentral compartment. Notably, CYP2E1 remained decreased even when quantified within the GS-defined pericentral compartment, suggesting that cytokine-driven repression and zonation-related remodeling likely act together. Spatially resolved analyses will be needed to define their relative contributions more precisely.

Fourth, because this transgenic model exhibits growth retardation and reduced survival, long-term tumor outcomes should be interpreted with caution. We also did not normalize tumor burden to liver weight. Although per-mouse metrics are widely used in DEN models, differences in liver mass could influence burden estimates and should be considered when interpreting group differences. Complementary approaches, such as adult-onset inducible genetic models or milder liver-injury settings, will help determine to what extent the observed phenotype is influenced by the systemic manifestations of this model.

Fifth, because DEN-induced hepatocarcinogenesis is strongly influenced by sex [17], we analyzed males exclusively. Nonetheless, explicitly positioning sex as a biological variable and considering validation frameworks that include females remain important future directions.

In addition, regulatory layers such as post-translational modification of nuclear receptor pathways (e.g., SUMOylation) may contribute to suppression of detoxification programs under inflammatory conditions [61,62], warranting integrative analyses of receptor activation states together with epigenomic profiling and transcriptional-machinery binding dynamics.

Taken together, our findings suggest that chronic inflammation can suppress pericentral xenobiotic-metabolism programs and thereby potentially limit genotoxic input during the initiation stage of chemical hepatocarcinogenesis. Direct measurements of DNA adducts and metabolic capacity, together with spatial analyses and complementary models, will be important for testing the causal mechanism proposed here.

## 5. Conclusions

This study shows that perturbation of hepatocyte IKKβ signaling can induce spontaneous chronic hepatitis and fibrosis with marked cell death and regenerative pressure while reducing the pericentral xenobiotic-metabolism program, particularly CYP2E1, a shift that would be expected to lessen DEN bioactivation and genotoxic stress. The Tg-IKKβ^Δhep^ model also extends our cell-culture observations by showing that nuclear IKKβ-dependent stress signaling can be recapitulated in hepatocytes in vivo. Overall, these findings are consistent with a metabolic-gatekeeping scenario in which perturbed hepatocyte IKKβ signaling promotes chronic hepatitis and fibrosis yet suppresses pericentral xenobiotic metabolism, thereby lowering DEN bioactivation and genotoxic input and, in turn, constraining tumor initiation. Defining how inflammatory signaling remodels hepatic zonation and detoxification networks through the HNF4α-PXR-CAR axis may inform risk stratification and prevention in inflammation-associated liver cancer.

## Data Availability

The microarray datasets generated in this study have been deposited in the NCBI Gene Expression Omnibus (GEO) under accession numbers GSE324983 and GSE324985. The ChIP-seq dataset generated in this study has been deposited in GEO under accession number GSE322777.

## Supplementary Materials

The following supporting information can be downloaded at: https://www.mdpi.com/article/10.3390/cells15060546/s1, Supplementary Figures S1–S11 and Supplementary Tables S1–S3 are available online. Supplementary Figure S1: Generation and validation of Tg-IKKβ^Δhep^ mice. Supplementary Figure S2: Gross liver appearance, liver-to-body weight ratio, and longitudinal serum ALT levels. Supplementary Figure S3: Representative staining and quantification of hepatic injury, oxidative stress, inflammation, and proliferation. Supplementary Figure S4: RT-qPCR analysis of fibrosis-related genes and inflammatory cytokines. Supplementary Figure S5: Additional analysis of γH2AX staining and glutamine synthetase distribution after DEN treatment. Supplementary Figure S6: Immunoblot analysis of CYP2E1, PGC-1α, and glutamine synthetase. Supplementary Figure S7: Transcriptome gene-set heatmaps in livers of the four genotypes (IKKβ^F/F^, IKKβ^Δhep^, Tg, and Tg-IKKβ^Δhep^). Supplementary Figure S8: GO enrichment analysis of differentially expressed genes in Tg-IKKβ^Δhep^ versus IKKβ^F/F^ livers. Supplementary Figure S9: Additional HNF4α staining fields, exploratory EMSA, and ChIP-qPCR analysis. Supplementary Figure S10: Exploratory microarray heatmaps after acute TNFα or IL-1β stimulation. Supplementary Figure S11: Representative Pol II ChIP-seq tracks at xenobiotic metabolism-related loci. Supplementary Table S1. Genotyping primers used in this study. Supplementary Table S2. Primer sequences used for RT-qPCR (grouped by functional). Supplementary Table S3. Oligonucleotide sequences used for EMSA and ChIP–qPCR.

## References

[1] Sung H., Ferlay J., Siegel R.L., Laversanne M., Soerjomataram I., Jemal A., Bray F. (2021). Global cancer statistics 2020: GLOBOCAN estimates of incidence and mortality worldwide for 36 cancers in 185 countries. CA Cancer J. Clin..

[2] El-Serag H.B., Rudolph K.L. (2007). Hepatocellular carcinoma: Epidemiology and molecular carcinogenesis. Gastroenterology.

[3] Llovet J.M., Kelley R.K., Villanueva A., Singal A.G., Pikarsky E., Roayaie S., Lencioni R., Koike K., Zucman-Rossi J., Finn R.S. (2021). Hepatocellular carcinoma. Nat. Rev. Dis. Primers.

[4] Forner A., Reig M., Bruix J. (2018). Hepatocellular carcinoma. Lancet.

[5] Rinella M.E., Lazarus J.V., Ratziu V., Francque S.M., Sanyal A.J., Kanwal F., Romero D., Shulman G.I., Caldwell S.H., Kowdley K.V. (2023). A multisociety Delphi consensus statement on new fatty liver disease nomenclature. J. Hepatol..

[6] Younossi Z.M., Koenig A.B., Abdelatif D., Fazel Y., Henry L., Wymer M. (2016). Global epidemiology of nonalcoholic fatty liver disease—Meta-analytic assessment of prevalence, incidence, and outcomes. Hepatology.

[7] Jungermann K., Kietzmann T. (2000). Oxygen: Modulator of metabolic zonation and disease of the liver. Hepatology.

[8] Oinonen T., Lindros K.O. (1998). Zonation of hepatic cytochrome P-450 expression and regulation. Biochem. J..

[9] Halpern K.B., Shenhav R., Matcovitch-Natan O., Tóth B., Lemze D., Golan M., Massasa E.E., Baydatch S., Landen S., Moor A.E. (2017). Single-cell spatial reconstruction reveals global division of labour in the mammalian liver. Nature.

[10] Gebhardt R., Matz-Soja M. (2014). Liver zonation: Novel aspects of its regulation and its impact on homeostasis. World J. Gastroenterol..

[11] Kietzmann T. (2017). Metabolic zonation of the liver: The oxygen gradient revisited. Redox Biol..

[12] Ang C.H., Hsu S.H., Guo F., Tan C.T., Yu V.C., Visvader J.E., Chow P.K.H., Fu N.Y. (2019). Lgr5+ pericentral hepatocytes are self-maintained in normal liver and expand in response to injury. Proc. Natl. Acad. Sci. USA.

[13] Kang J.S., Wanibuchi H., Morimura K., Gonzalez F.J., Fukushima S. (2007). Role of CYP2E1 in diethylnitrosamine-induced hepatocarcinogenesis in vivo. Cancer Res..

[14] Guengerich F.P. (2020). Cytochrome P450 2E1 and its roles in disease. Chem. Biol. Interact..

[15] Gao J., Wang Z., Wang G.J., Zhang H.X., Gao N., Wang J., Zhang Y.F., Ma J.M., Wang X.M., Qian Y.M. (2018). Higher CYP2E1 activity correlates with hepatocarcinogenesis induced by diethylnitrosamine. J. Pharmacol. Exp. Ther..

[16] Maeda S., Kamata H., Luo J.L., Leffert H., Karin M. (2005). IKKβ couples hepatocyte death to cytokine-driven compensatory proliferation that promotes chemical hepatocarcinogenesis. Cell.

[17] Naugler W.E., Sakurai T., Kim S., Maeda S., Kim K., Elsharkawy A.M., Karin M. (2007). Gender disparity in liver cancer due to sex differences in MyD88-dependent IL-6 production. Science.

[18] Pikarsky E., Porat R.M., Stein I., Abramovitch R., Amit S., Kasem S., Gutkovich-Pyest E., Urieli-Shoval S., Galun E., Ben-Neriah Y. (2004). NF-κB functions as a tumour promoter in inflammation-associated cancer. Nature.

[19] Dhar D., Antonucci L., Nakagawa H., Kim J.Y., Glitzner E., Caruso S., Shalapour S., Yang L., Valasek M.A., Lee S. (2018). Liver cancer initiation requires p53 inhibition by CD44-enhanced growth factor signaling. Cancer Cell.

[20] Sakurai T., Maeda S., Chang L., Karin M. (2006). Loss of hepatic NF-κB activity enhances chemical hepatocarcinogenesis through sustained c-Jun N-terminal kinase 1 activation. Proc. Natl. Acad. Sci. USA.

[21] Kamata H., Honda S., Maeda S., Chang L., Hirata H., Karin M. (2005). Reactive oxygen species promote TNFalpha-induced death and sustained JNK activation by inhibiting MAP kinase phosphatases. Cell.

[22] Luedde T., Beraza N., Kotsikoris V., van Loo G., Nenci A., De Vos R., Roskams T., Trautwein C., Pasparakis M. (2007). Deletion of NEMO/IKKγ in liver parenchymal cells causes steatohepatitis and hepatocellular carcinoma. Cancer Cell.

[23] Kondylis V., Polykratis A., Ehlken H., Ochoa-Callejero L., Straub B.K., Krishna-Subramanian S., Van T.-M., Curth H.-M., Heise N., Weih F. (2015). NEMO prevents steatohepatitis and hepatocellular carcinoma by inhibiting RIPK1 kinase activity-mediated hepatocyte apoptosis. Cancer Cell.

[24] Haybaeck J., Zeller N., Wolf M.J., Weber A., Wagner U., Kurrer M.O., Bremer J., Iezzi G., Graf R., Clavien P.-A. (2009). A lymphotoxin-driven pathway to hepatocellular carcinoma. Cancer Cell.

[25] He G., Yu G.Y., Temkin V., Ogata H., Kuntzen C., Sakurai T., Sieghart W., Peck-Radosavljevic M., Leffert H.L., Karin M. (2010). Hepatocyte IKKβ/NF-κB inhibits tumor promotion and progression by preventing oxidative stress-driven STAT3 activation. Cancer Cell.

[26] Grivennikov S.I., Greten F.R., Karin M. (2010). Immunity, inflammation, and cancer. Cell.

[27] Tsuchiya Y., Asano T., Nakayama K., Kato T., Karin M., Kamata H. (2010). Nuclear IKKβ is an adaptor protein for IκBα ubiquitination and degradation in UV-induced NF-κB activation. Mol. Cell.

[28] Willson T.M., Kliewer S.A. (2002). PXR, CAR and drug metabolism. Nat. Rev. Drug Discov..

[29] Lehmann J.M., McKee D.D., Watson M.A., Willson T.M., Moore J.T., Kliewer S.A. (1998). The human orphan nuclear receptor PXR is activated by compounds that regulate CYP3A4 gene expression and cause drug interactions. J. Clin. Investig..

[30] Morgan E.T. (2001). Regulation of cytochrome P450 by inflammatory mediators: Why and how?. Drug Metab. Dispos..

[31] Morgan E.T. (2009). Impact of infectious and inflammatory disease on cytochrome P450-mediated drug metabolism and pharmacokinetics. Clin. Pharmacol. Ther..

[32] Constandinou C., Henderson N., Iredale J.P. (2005). Modeling liver fibrosis in rodents. Methods Mol. Med..

[33] Ye Q., Lian F., Chavez P.R.G., Chung J., Ling W., Beretta L. (2012). Cytochrome P450 2E1 inhibition prevents hepatic carcinogenesis induced by diethylnitrosamine in alcohol-fed rats. Hepatobiliary Surg. Nutr..

[34] Parviz F., Matullo C., Garrison W.D., Savatski L., Adamson J.W., Ning G., Kaestner K.H., Rossi J.M., Zaret K.S., Duncan S.A. (2000). Mammalian hepatocyte differentiation requires the transcription factor HNF-4α. Genes Dev..

[35] Hayhurst G.P., Lee Y.H., Lambert G., Ward J.M., Gonzalez F.J. (2001). Hepatocyte nuclear factor 4α (HNF4α) is essential for maintenance of hepatic gene expression and lipid homeostasis. Mol. Cell. Biol..

[36] Walesky C., Edwards G., Borude P., Gunewardena S., O’Neil M., Yoo B., Apte U. (2013). Hepatocyte nuclear factor 4 alpha deletion promotes diethylnitrosamine-induced hepatocellular carcinoma in rodents. Hepatology.

[37] Yli-Peltola K., Määttä J.A., Huovari J., Miettinen P.J. (2012). Suppression of hepatocyte proliferation by hepatocyte nuclear factor 4α in adult mice. J. Biol. Chem..

[38] Ning B.-F., Ding J., Yin C., Zhong W., Wu K., Zeng X., Yang W., Chen Y.-X., Zhang J.-P., Zhang X. (2010). Hepatocyte nuclear factor 4 alpha suppresses the development of hepatocellular carcinoma. Cancer Res..

[39] Ning B.F., Ding J., Liu J., Yin C., Xu W.P., Cong W.M., Zhang Q., Guo R.H., Wang Y., Wang L. (2014). HNF4α-NF-κB feedback circuit modulates liver cancer progression. Hepatology.

[40] Hatziapostolou M., Polytarchou C., Aggelidou E., Drakaki A., Poultsides G.A., Jaeger S.A., Ogata H., Karin M., Struhl K., Hadzopoulou-Cladaras M. (2011). An HNF4α-miRNA inflammatory feedback circuit regulates hepatocellular oncogenesis. Cell.

[41] Shin D.-J., Campos J.A., Gil G., Osborne T.F. (2003). Peroxisome proliferator-activated receptor gamma coactivator-1α activates CYP7A1 and bile acid biosynthesis. J. Biol. Chem..

[42] Miao J., Fang S., Bae Y., Kemper J.K. (2006). Functional inhibitory cross-talk between constitutive androstane receptor and hepatic nuclear factor-4 in hepatic lipid/glucose metabolism is mediated by competition for binding to the DR1 motif and to the common coactivators, GRIP-1 and PGC-1α. J. Biol. Chem..

[43] Léveillé M., Besse-Patin A., Dufour C.R., Gagnon A., Julienne H., Côté M., St-Pierre J., Estall J.L. (2020). PGC-1α isoforms coordinate to balance hepatic metabolism and apoptosis in inflammatory environments. Mol. Metab..

[44] Huck I., Gunewardena S., Espanol-Suner R., Willenbring H., Apte U. (2019). Hepatocyte nuclear factor 4 alpha activation is essential for termination of liver regeneration in mice. Hepatology.

[45] Li T., Jahan A., Chiang J.Y.L. (2006). Bile acids and cytokines inhibit the human cholesterol 7 alpha-hydroxylase gene via the JNK/c-Jun pathway in human liver cells. Hepatology.

[46] Benhamouche S., Decaens T., Godard C., Chambrey R., Rickman D.S., Moinard C., Vasseur-Cognet M., Kuo C.J., Kahn A., Perret C. (2006). Apc tumor suppressor gene is the “zonation-keeper” of mouse liver. Dev. Cell.

[47] Burke Z.D., Reed K.R., Phesse T.J., Sansom O.J., Clarke A.R., Tosh D. (2009). Liver zonation occurs through a β-catenin-dependent, c-Myc-independent mechanism. Gastroenterology.

[48] Gerbal-Chaloin S., Dumé A.S., Briolotti P., Klieber S., Raulet E., Duret C., Fabre J.-M., Ramos J., Maurel P., Daujat-Chavanieu M. (2014). The WNT/β-catenin pathway is a transcriptional regulator of CYP2E1, CYP1A2, and aryl hydrocarbon receptor gene expression in primary human hepatocytes. Mol. Pharmacol..

[49] Zhang X.-F., Tan X., Zeng G., Misse A., Singh S., Kim Y., Klaunig J.E., Monga S.P.S. (2010). Conditional beta-catenin loss in mice promotes chemical hepatocarcinogenesis: Role of oxidative stress and platelet-derived growth factor receptor alpha/phosphoinositide 3-kinase signaling. Hepatology.

[50] Rignall B., Braeuning A., Buchmann A., Schwarz M. (2011). Tumor formation in liver of conditional beta-catenin-deficient mice exposed to a diethylnitrosamine/phenobarbital tumor promotion regimen. Carcinogenesis.

[51] Assenat E., Gerbal-Chaloin S., Larrey D., Saric J., Fabre J.-M., Maurel P., Vilarem M.-J., Pascussi J.-M. (2004). Interleukin 1β inhibits CAR-induced expression of hepatic genes involved in drug and bilirubin clearance. Hepatology.

[52] Zhou C., Tabb M.M., Nelson E.L., Grün F., Verma S., Sadatrafiei A., Lin M., Mallick S., Forman B.M., Thummel K.E. (2006). Mutual repression between steroid and xenobiotic receptor and NF-kappaB signaling pathways links xenobiotic metabolism and inflammation. J. Clin. Investig..

[53] Anstee Q.M., Reeves H.L., Kotsiliti E., Govaere O., Heikenwalder M. (2019). From NASH to HCC: Current concepts and future challenges. Nat. Rev. Gastroenterol. Hepatol..

[54] Fekry B., Ribas-Latre A., Baumgartner C., Mohamed A., Kolonin M.G., Sladek F.M., Younes M., Eckel-Mahan K.L. (2019). HNF4α-deficient fatty liver provides a permissive environment for sex-independent hepatocellular carcinoma. Cancer Res..

[55] Gu L., Zhu Y., Nandi S.P., Lee M., Watari K., Bareng B., Manna S., Singhal A., Yopp A.C., Mizukami Y. (2025). FBP1 controls liver cancer evolution from senescent MASH hepatocytes. Nature.

[56] Ramadass V., Vaiyapuri T., Tergaonkar V. (2020). Small molecule NF-κB pathway inhibitors in clinic. Int. J. Mol. Sci..

[57] Oneta C.M., Lieber C.S., Li J., Rüttimann S., Schmid B., Lattmann J., Rosman A.S., Seitz H.K. (2002). Dynamics of cytochrome P4502E1 activity in man: Induction by ethanol and disappearance during withdrawal phase. J. Hepatol..

[58] Aubert J., Begriche K., Knockaert L., Robin M.-A., Fromenty B. (2011). Increased expression of cytochrome P450 2E1 in nonalcoholic fatty liver disease: Mechanisms and pathophysiological role. Clin. Res. Hepatol. Gastroenterol..

[59] Churchwell M.I., Beland F.A., Doerge D.R. (2006). Quantification of O6-methyl and O6-ethyl deoxyguanosine adducts in C57BL/6N/Tk+/− mice using LC/MS/MS. J. Chromatogr. B Analyt. Technol. Biomed. Life Sci..

[60] Balbo S., Turesky R.J., Villalta P.W. (2014). DNA adductomics. Chem. Res. Toxicol..

[61] Hu G., Xu C., Staudinger J.L. (2010). Pregnane X receptor is SUMOylated to repress the inflammatory response. J. Pharmacol. Exp. Ther..

[62] Staudinger J.L. (2011). Post-translational modification of pregnane X receptor. Pharmacol. Res..

