# Supplementary material for: Inflammation-Driven Downregulation of CYP2E1 Is Associated with Attenuated Diethylnitrosamine (DEN)-Induced Hepatocarcinogenesis
Source: Cells. 2026 Mar 19;15(6):546. doi: 10.3390/cells15060546 (PMC13025445; doi:10.3390/cells15060546)
Supplement: Supplementary file 1 [file cells-15-00546-s001.zip › Supplementary Figure Legends S1-S11 final.pdf]

Supplementary Figure Legends

Supplementary Figure S1

(A) Transgene design. Schematic of the FLAG–NLS–IKKβ (K44A) expression cassette. Expression is driven by a ~4.0 kb mouse Ikbkb 5′ genomic regulatory fragment (−4000 to −1 relative to the translation start codon; including non-coding exon(s)/intron(s)). The cassette encodes an N-terminal FLAG tag followed by a nuclear localization signal (NLS) and mouse IKKβ (Ikbkb) carrying the kinase-inactivating K44A mutation, followed by a polyadenylation (polyA) signal. The transgene was introduced by pronuclear injection and randomly integrated into the genome; the insertion site and copy number were not determined. (B) Breeding scheme to generate Tg-IKKβ$^{\Delta hep}$ mice. Tg mice (genotype: Tg; IKKβ$^{F/F}$; Alb-Cre^{-}) were crossed with hepatocyte-specific IKKβ conditional knockout mice (genotype: IKKβ$^{F/F}$; Alb-Cre^{+}; hereafter IKKβ$^{\Delta hep}$) to obtain four littermate genotypes (control: IKKβ$^{F/F}$; Alb-Cre^{-}; Tg^{-}, IKKβ$^{\Delta hep}$: IKKβ$^{F/F}$; Alb-Cre^{+}; Tg^{-}, Tg: Tg; IKKβ$^{F/F}$; Alb-Cre^{-}, and Tg-IKKβ$^{\Delta hep}$: Tg; IKKβ$^{F/F}$; Alb-Cre^{+}). Unless otherwise noted, littermates were used as controls. (C) Immunoblot validation of hepatic transgene expression. Whole-cell lysates from IKKβ$^{F/F}$, Tg, and Tg-IKKβ$^{\Delta hep}$ livers were immunoblotted with an anti-IKKβ antibody. The upper, slower-migrating band corresponds to the transgene product (FLAG–NLS–IKKβ (K44A)), whereas the lower band represents endogenous IKKβ. β-actin served as the loading control (IKKβ$^{F/F}$: #1, #4, #7, #10; Tg: #2, #5, #8, #11; Tg-IKKβ$^{\Delta hep}$: #3, #6, #9, #12). (D) Immunoblot validation of hepatic transgene expression in subcellular fractions. Cytoplasmic and nuclear fractions were prepared from Tg-IKKβ$^{\Delta hep}$ livers (founder lines #3, #6, #9, and #12) using the Nuclear/Cytosol Fractionation Kit (BioVision) according to the manufacturer's instructions. The transgene was detected using anti-FLAG and/or anti-IKKβ antibodies. α-Tubulin and Lamin B1 were used as markers for cytoplasmic and nuclear fractions, respectively. (E) Representative gross appearance. Representative photographs of 12-week-old male mice (left: control/IKKβ$^{F/F}$; right: Tg-IKKβ$^{\Delta hep}$). Tg-IKKβ$^{\Delta hep}$ mice displayed clear growth retardation compared with control littermates under identical housing and feeding conditions.

Supplementary Figure S2

(A) Representative gross liver images from the indicated genotypes at 4 and 16 weeks of age. (B) Liver-to-body weight ratio at 16 weeks of age. Panel B was analyzed using an ordinary one-way ANOVA followed by Dunnett's multiple comparisons test (Tg-IKKβ$^{\Delta hep}$ as the control). Mean liver-to-body weight ratio was 0.08338 in Tg-IKKβ$^{\Delta hep}$ vs 0.03963 in IKKβ$^{F/F}$ (mean diff. = 0.04375, 95% CI, 0.03111 to 0.05639), vs 0.03513 in IKKβ$^{\Delta hep}$ (mean diff. = 0.04825, 95% CI, 0.03561 to 0.06089), and vs 0.03550 in Tg (mean diff. = 0.04788, 95% CI, 0.03524 to 0.06051). (C) Longitudinal serum ALT measurements. Longitudinal serum ALT measurements at 4 (n = 6), 16 (n = 5), 36 (n = 4), and 48 (n = 4) weeks of age in IKKβ$^{F/F}$ and Tg-IKKβ$^{\Delta hep}$ mice. Panel C was analyzed using an ordinary one-way ANOVA followed by Dunnett's multiple comparisons test (IKKβ$^{F/F}$ as the control) to compare Tg-IKKβ$^{\Delta hep}$ vs IKKβ$^{F/F}$ at each time point. At 4 weeks, mean IKKβ$^{F/F}$ = 19.07 U/L and mean Tg-IKKβ$^{\Delta hep}$ = 390.6 U/L (difference = -371.5, SE = 40.76, p = 0.000004); at 16 weeks, 15.94 vs 223.1 U/L (difference = -207.2, SE = 26.49, p = 0.000051); at 36 weeks, 16.51 vs 103.6 U/L (difference = -87.12, SE = 9.204, p = 0.000079); and at 48 weeks, 21.78 vs 77.96 U/L (difference = -56.18, SE = 5.592, p = 0.000056). For panel C, multiple unpaired, two-tailed t-tests were also performed (as reported in the Prism output). Data are presented as mean ± SEM unless otherwise stated. * $p < 0.05$, **** $p < 0.0001$.

Supplementary Figure S3

Representative staining of liver sections from 4-week-old mice. (A) Immunostaining for RelA and immunofluorescence staining for 8-OHdG (scale bar: 50 μm). Immunofluorescence staining for TUNEL, F4/80, Ki67, and phospho-c-Jun (Ser63) (scale bar: 100 μm). (B) Quantification (left to right) of TUNEL-, Ki67-, 8-OHdG-, and phospho-c-Jun–positive cells (*n* = 4 per group). For immunofluorescence quantification, at least four randomly selected fields were analyzed per mouse. Statistical analysis was performed using an ordinary one-way ANOVA followed by Dunnett's multiple comparisons test (vs. $IKK\beta^{F/F}$), unless otherwise noted. Data are presented as mean ± SEM unless otherwise stated. ** $p < 0.01$, *** $p < 0.001$, **** $p < 0.0001$.

Supplementary Figure S4

RT-qPCR analysis of fibrosis-related genes and inflammatory cytokines in liver tissues from 6-week-old mice of the four genotypes: $IKK\beta^{F/F}$ (*n* = 3), $IKK\beta^{\Delta hep}$ (*n* = 3), Tg (*n* = 3), and Tg-$IKK\beta^{\Delta hep}$ (*n* = 4). Data are presented as mean ± SEM. Statistical analysis was performed using one-way ANOVA with Dunnett's multiple-comparison test (vs. $IKK\beta^{F/F}$). ** $p < 0.01$, *** $p < 0.001$, **** $p < 0.0001$.

Supplementary Figure S5

(A) Representative immunostaining for phosphorylated H2AX (Ser139) in liver sections at 48 h after DEN administration (50 mg/kg, i.p.) in 8-week-old mice. Enlarged views of the pericentral region defined by glutamine synthetase (GS)-positive hepatocytes are shown. Scale bar: 100 μm. Upper panels indicate untreated controls. (B) GS mean gray value (a.u.) and GS-positive area (%) were quantified in the indicated regions. Data are presented as mean ± SEM (*n* = 4), with each dot representing one mouse. Statistical significance was determined using an unpaired two-tailed Student's t-test. * $p < 0.05$.

Supplementary Figure S6

(A) Immunoblot analysis of liver extracts from 6-week-old mice for CYP2E1, PGC-1α and glutamine synthetase (GS); β-actin served as a loading control (*n* = 3 per group). (B) Densitometric quantification. Densitometric quantification of CYP2E1, PGC-1α, and GS normalized to β-actin. Data are presented as mean ± SEM unless otherwise stated. Statistical analysis was performed using an ordinary one-way ANOVA followed by Dunnett's multiple-comparisons test (vs. $IKK\beta^{F/F}$). * $p < 0.05$, ** $p < 0.01$.

Supplementary Figure S7

Transcriptome gene-set heatmaps demonstrate coordinated suppression of xenobiotic metabolism and mitochondrial respiration programs in livers from 6-week-old mice of the Tg-IKKβ$^{\Delta hep}$ genotype. (A) Heatmap of curated zone 3 Cyp genes involved in xenobiotic and bile acid metabolism. (B) Heatmap of mitochondrial respiration/OXPHOS-related genes. (C) Heatmap of representative transcriptional regulators, including the HNF4α–PGC-1α axis and nuclear receptor pathways. (D) Heatmap of inflammatory and extracellular matrix (ECM) remodeling markers. Expression values are shown as Z-scores of log2-transformed expression across the four genotypes (IKKβ$^{F/F}$, IKKβ$^{\Delta hep}$, Tg, and Tg-IKKβ$^{\Delta hep}$).

Supplementary Figure S8

GO enrichment analysis of Tg-IKKβ$^{\Delta hep}$ vs IKKβ$^{F/F}$. (A) Top GO terms enriched among genes upregulated in Tg-IKKβ$^{\Delta hep}$ livers. (B) Top GO terms enriched among genes downregulated in Tg-IKKβ$^{\Delta hep}$ livers. GO analysis was performed with multiple-testing correction. Bars indicate −log10(FDR). Annotations at the bar ends indicate fold enrichment (FE) and the number of genes contributing to each term.

Supplementary Figure S9

(A) Additional fields of view corresponding to the HNF4α (red) immunofluorescence staining shown in Figure 4B (DAPI, blue). Scale bar: 100 µm. (B) Exploratory EMSA. Exploratory EMSA using an HNF4α-binding sequence (Cyp8b1) suggests a trend toward reduced HNF4α DNA-binding activity in Tg-IKKβ$^{\Delta hep}$ liver extracts (n = 1 per condition). Arrows indicate HNF4α–DNA complexes. (C) Pol II and HNF4α ChIP-qPCR. ChIP-qPCR analysis of promoter occupancy of RNA polymerase II (Pol II) and HNF4α at the Cyp8b1 and Nr1i3 (CAR) promoters in IKKβ$^{F/F}$ and Tg-IKKβ$^{\Delta hep}$ livers. Data in panel C are presented as mean ± SEM. Statistical analysis was performed using an unpaired, two-tailed Student's *t*-test. * $p < 0.05$.

Supplementary Figure S10

Exploratory microarray heatmaps. Acute TNFα/IL-1β stimulation induces inflammatory programs and suppresses metabolic gene expression in wild-type (WT) livers (exploratory microarray dataset). (A) Heatmap of curated zone 3 Cyp genes involved in xenobiotic and bile acid metabolism. (B) Heatmap of mitochondrial respiration/OXPHOS-related genes. (C) Heatmap of transcriptional regulators, including the HNF4α–PGC-1α axis and representative nuclear receptor pathways. (D) Heatmap of inflammatory and ECM remodeling markers. Each heatmap summarizes exploratory microarray data from livers of 8-week-old male wild-type (WT) C57BL/6J mice collected 4 h after saline, TNFα, or IL-1β administration (n = 1 per condition). Key transcriptional changes were validated by RT-qPCR (Figure 5B).

Supplementary Figure S11

Representative Pol II ChIP-seq tracks. Representative RNA polymerase II (Pol II) ChIP-seq tracks at xenobiotic metabolism–related loci. Genome browser views show Pol II occupancy across the Cyp2e1, Cyp7a1, and Cyp8b1 loci in livers from 6-week-old male WT (control), WT (TNFα, 4 h), and Tg-IKKβ$^{\Delta hep}$ mice. Because the absolute ChIP-seq signal tends to be low at the Cyp7a1 and Cyp8b1 loci, changes in Pol II occupancy at these loci were additionally validated by Pol II ChIP-qPCR (Figure 5C).
