# Supplementary material for: Inflammation-Driven Downregulation of CYP2E1 Is Associated with Attenuated Diethylnitrosamine (DEN)-Induced Hepatocarcinogenesis
Source: Cells. 2026 Mar 19;15(6):546. doi: 10.3390/cells15060546 (PMC13025445; doi:10.3390/cells15060546)

Supplementary Figure S1

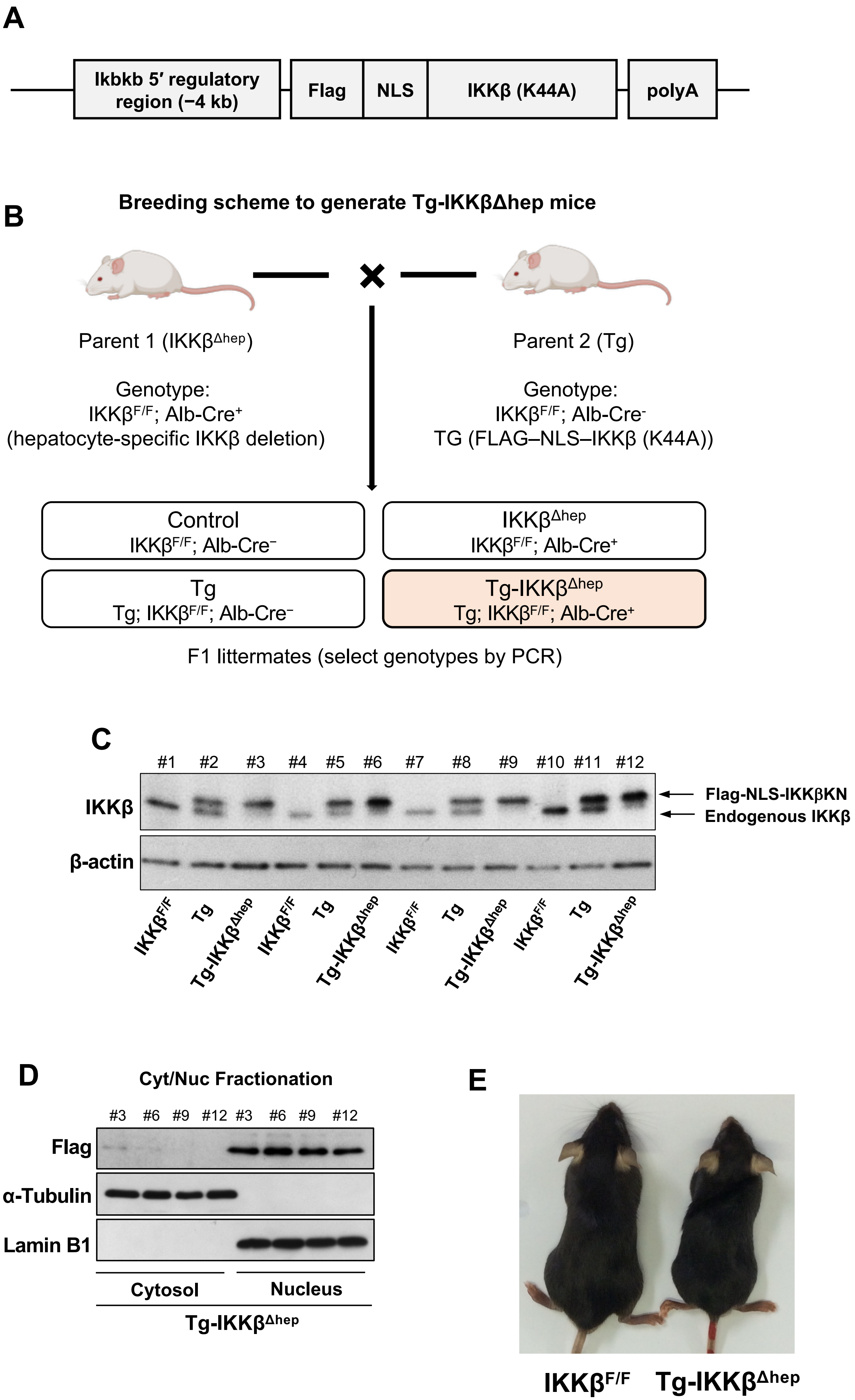

Supplementary Figure S2

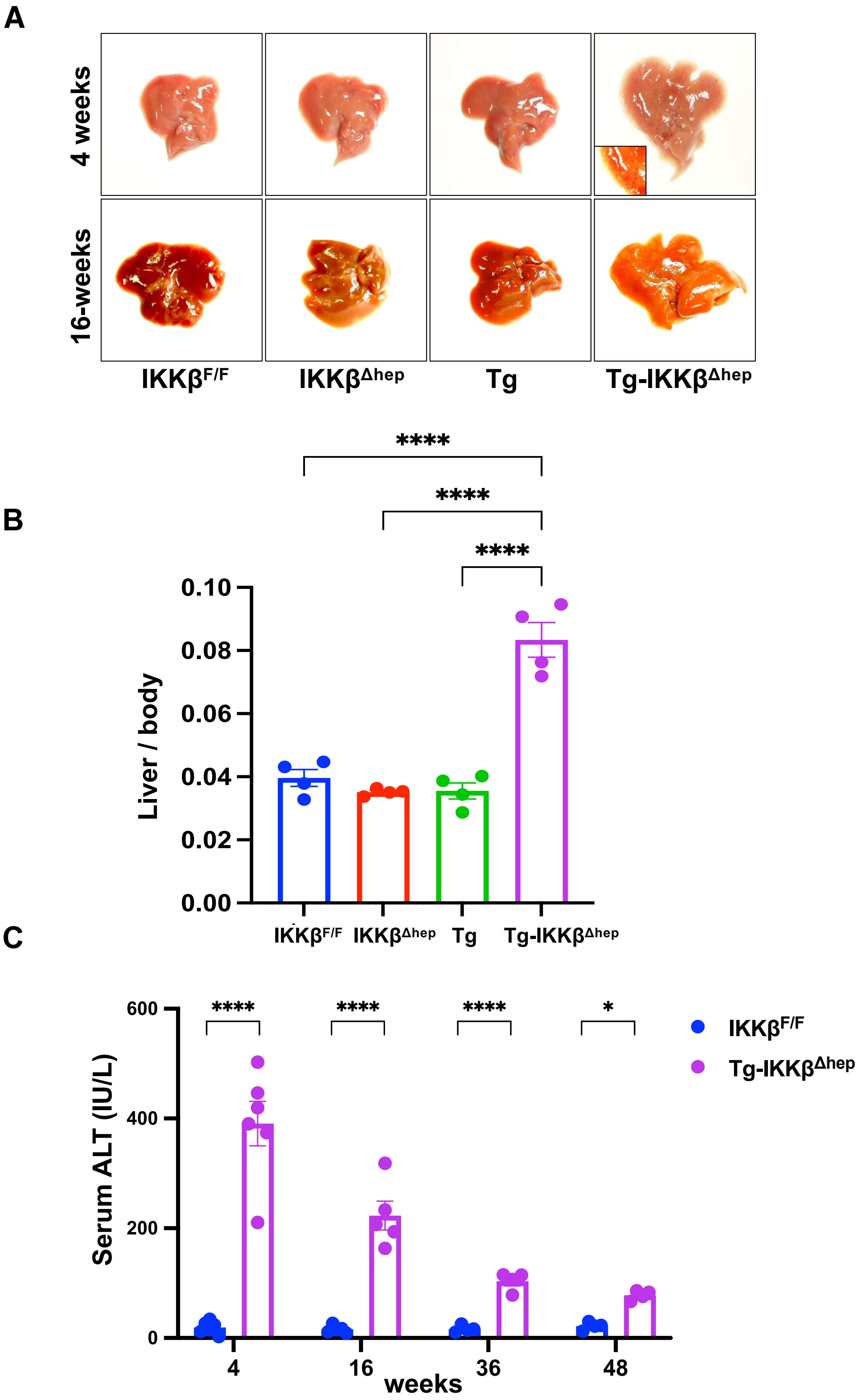

Supplementary Figure S3

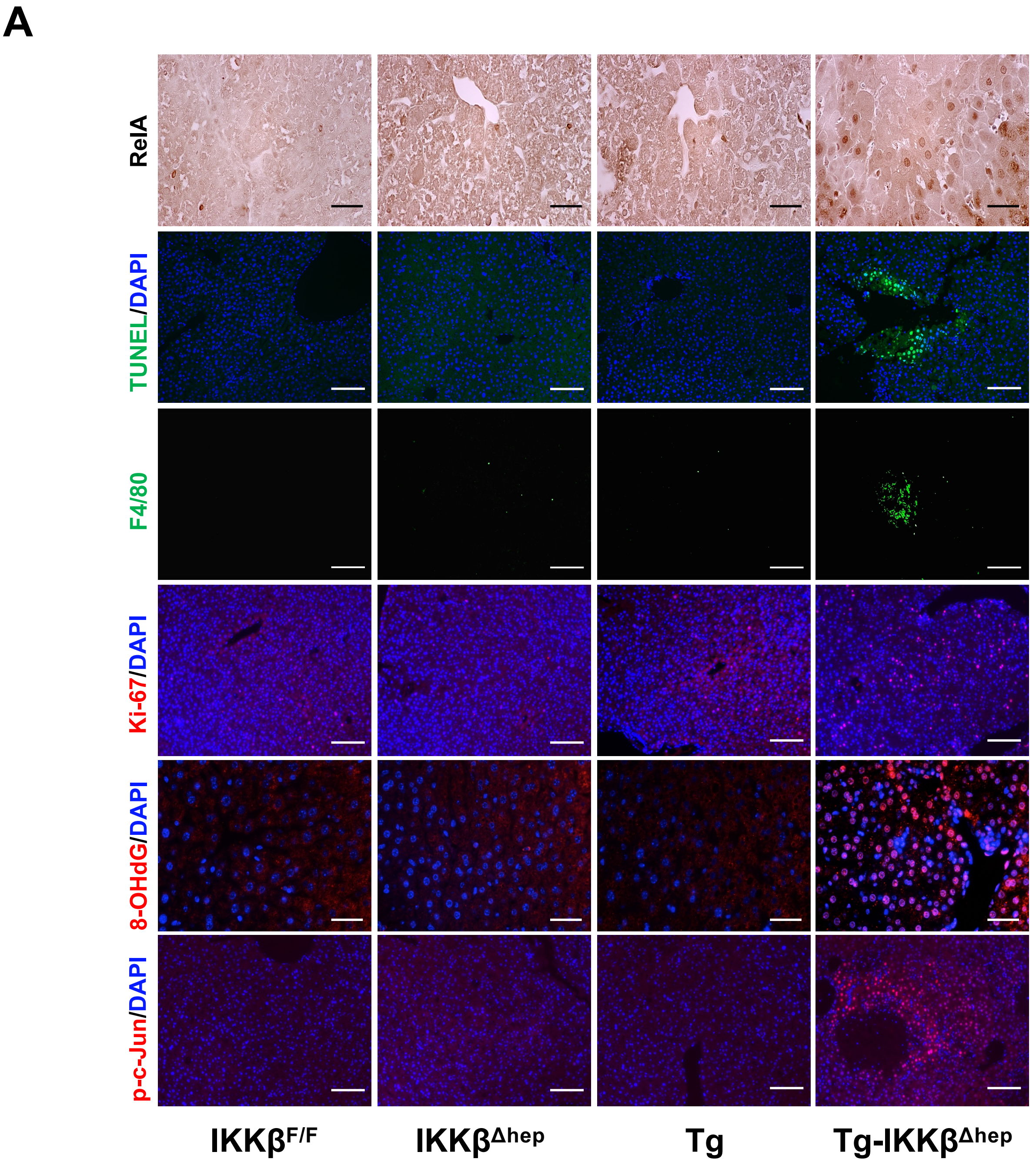

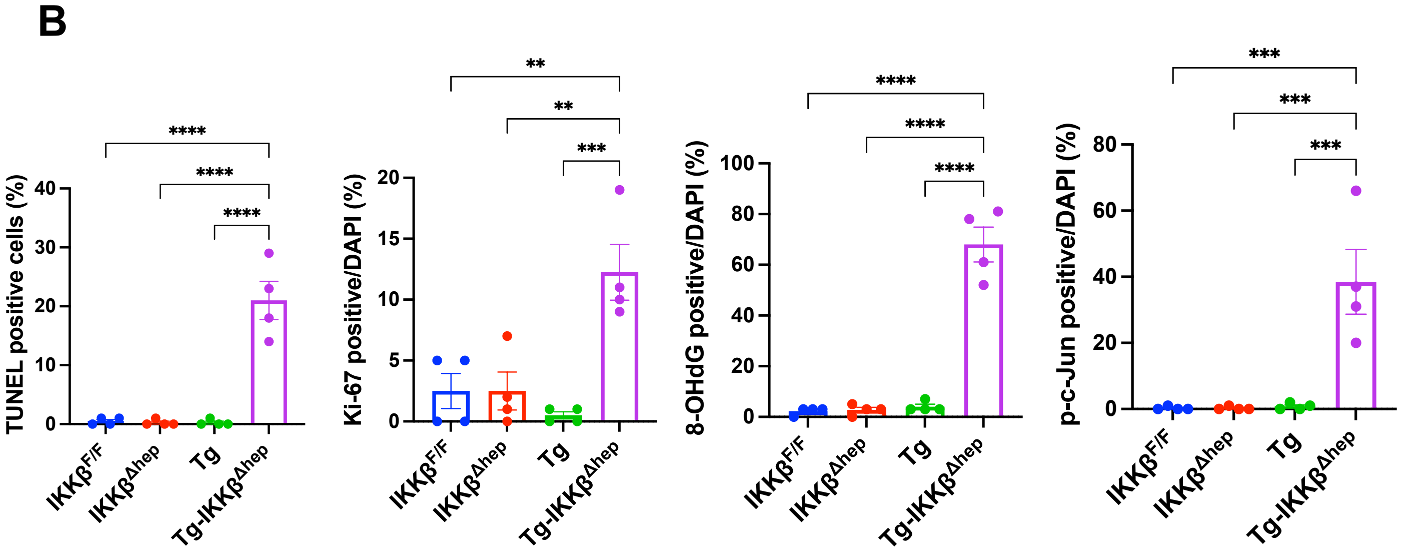

Supplementary Figure S4

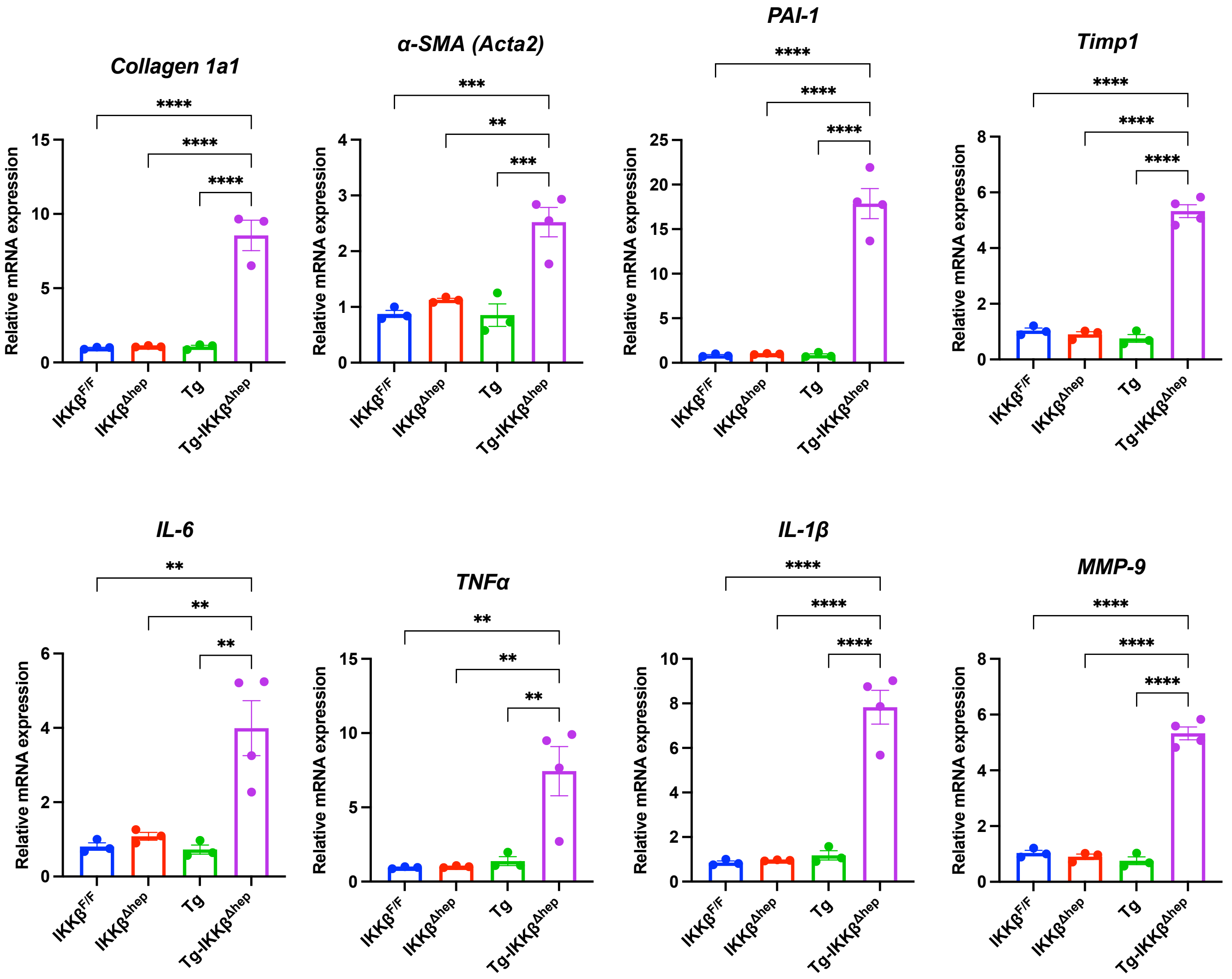

Supplementary Figure S5

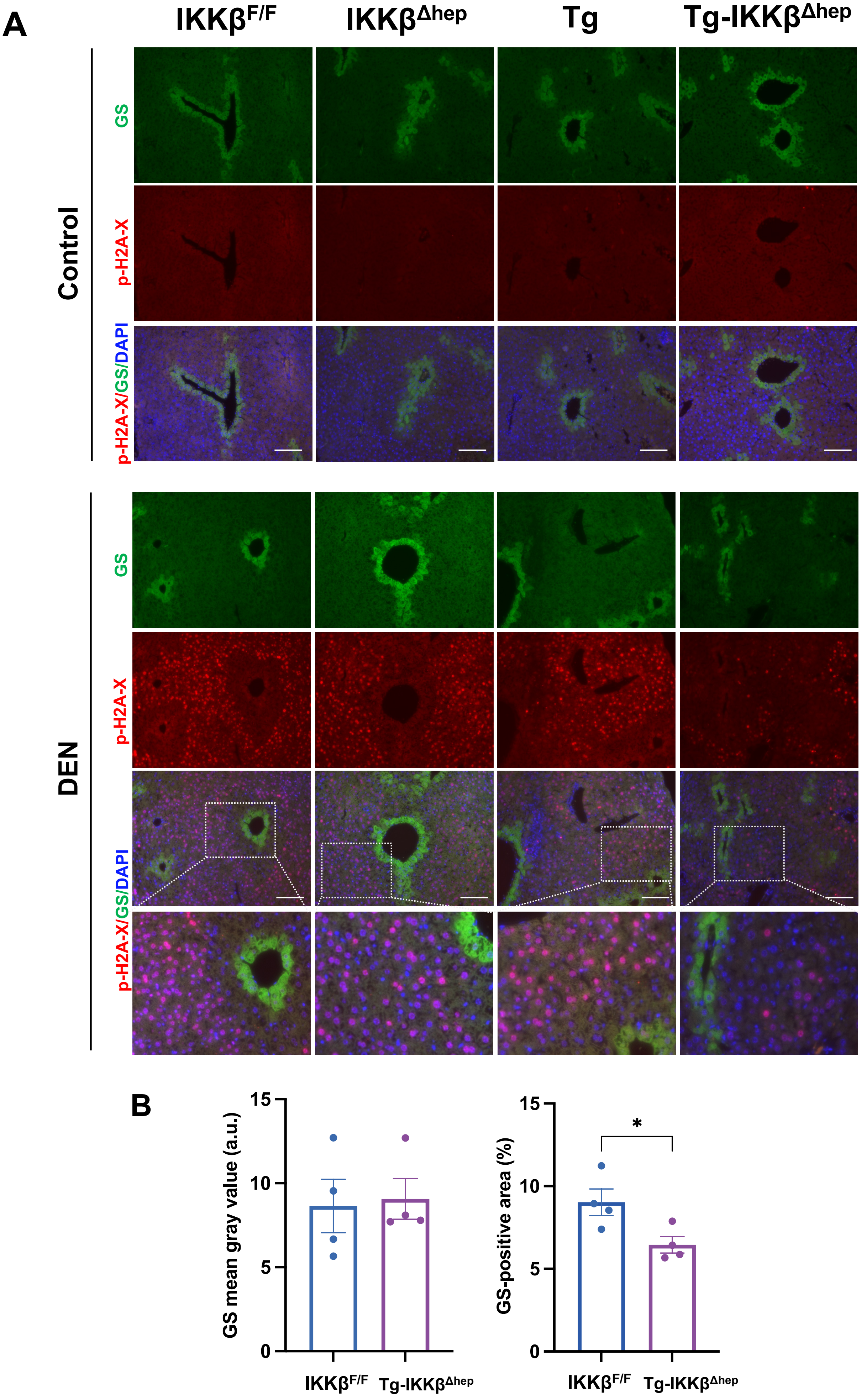

Supplementary Figure S6

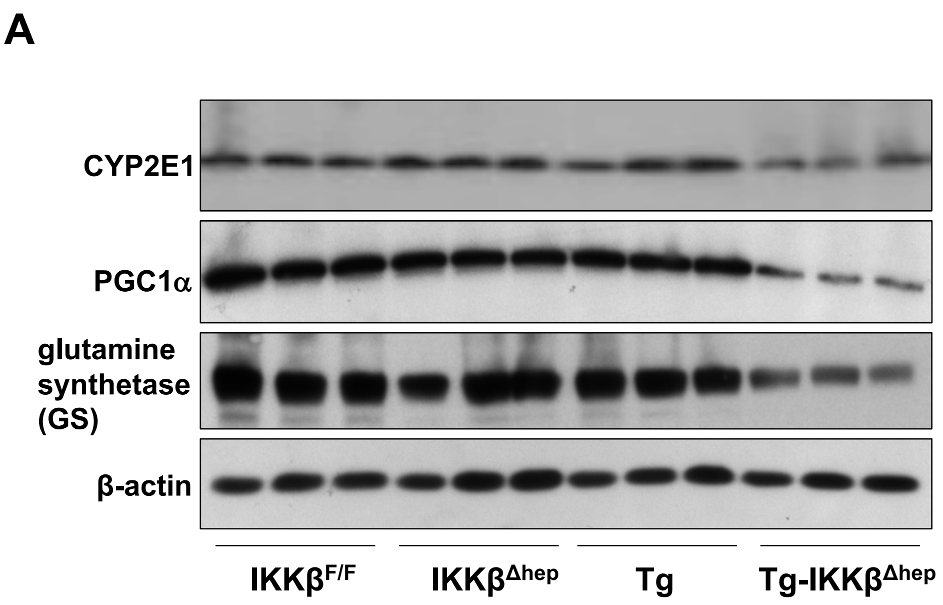

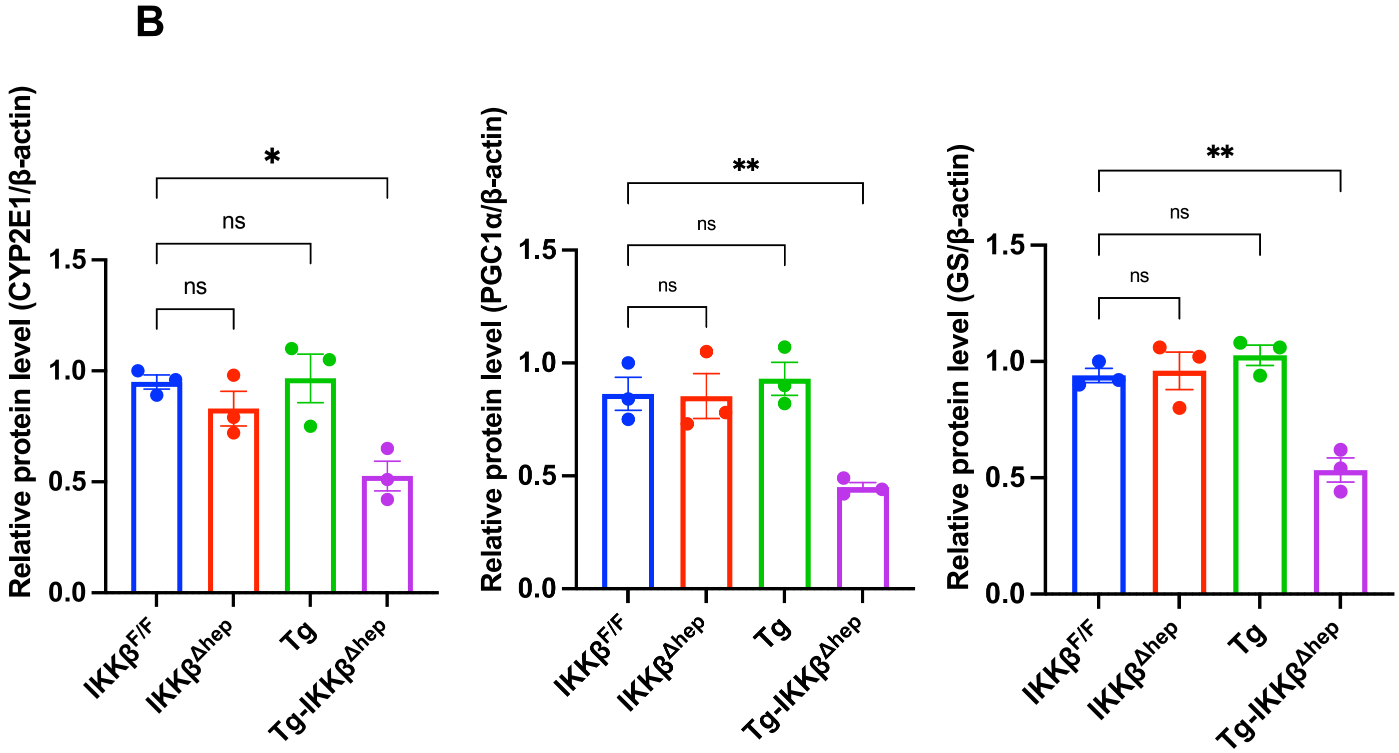

Supplementary Figure S7

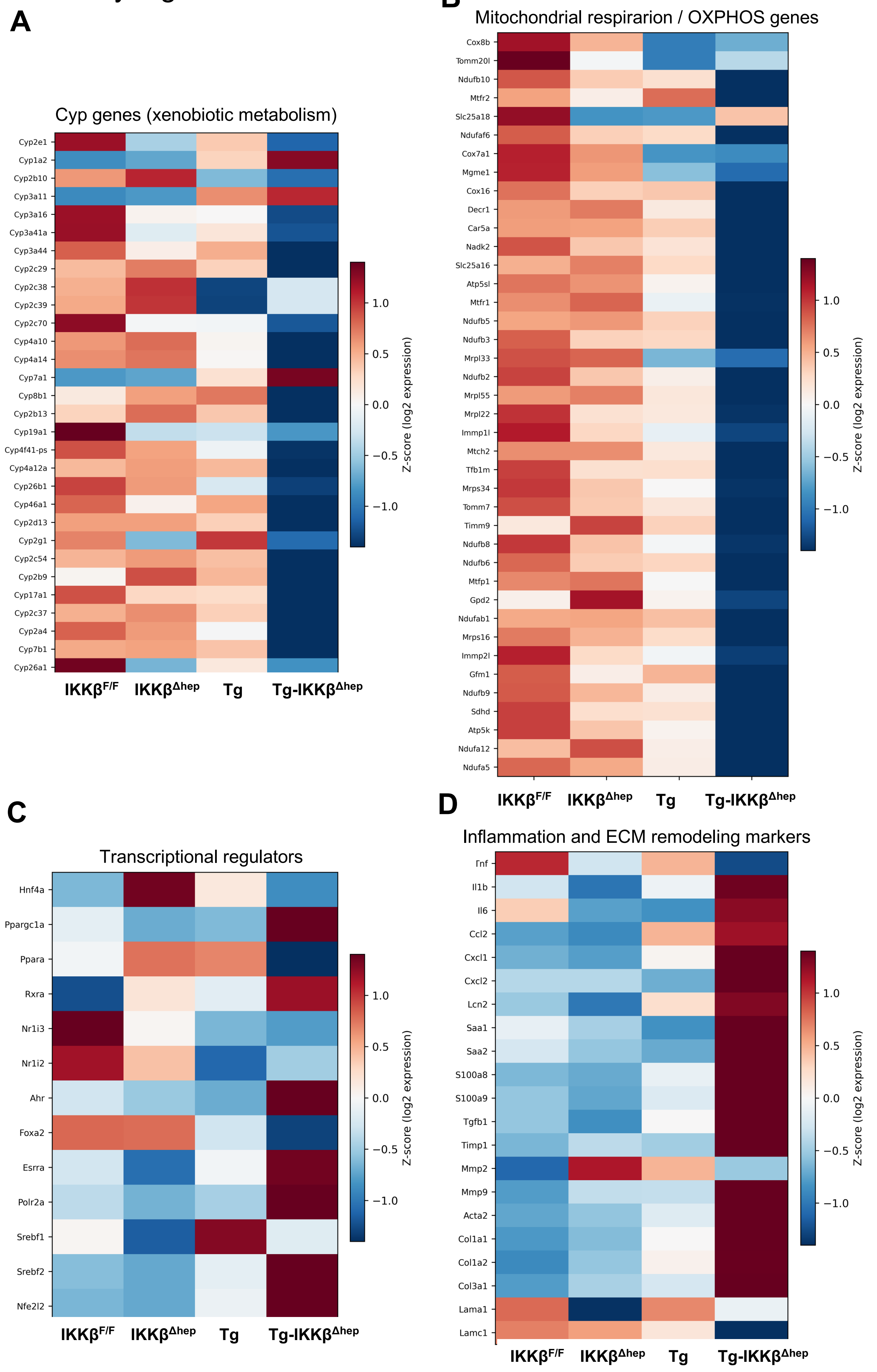

Supplementary Figure S8

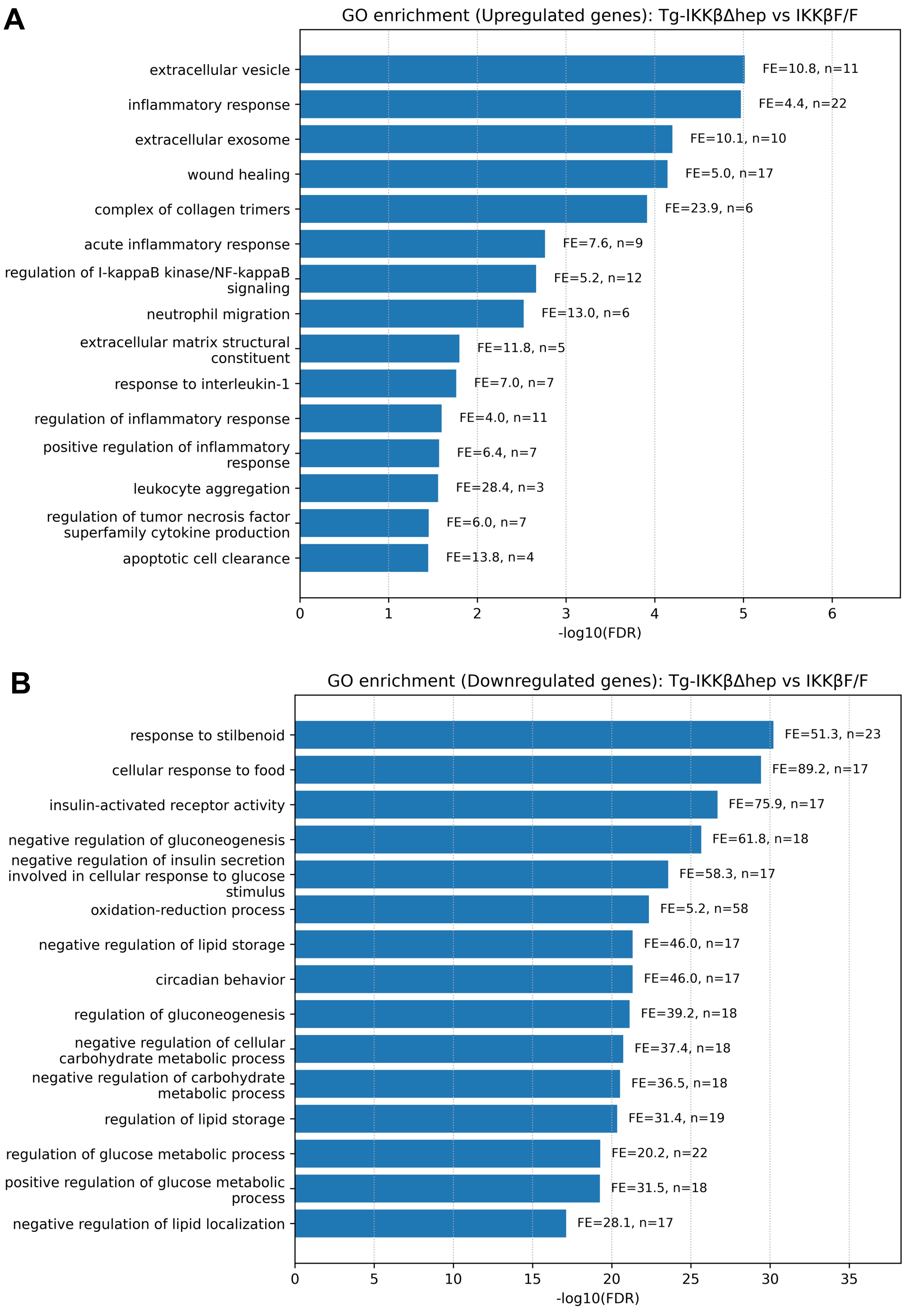

Supplementary Figure S9

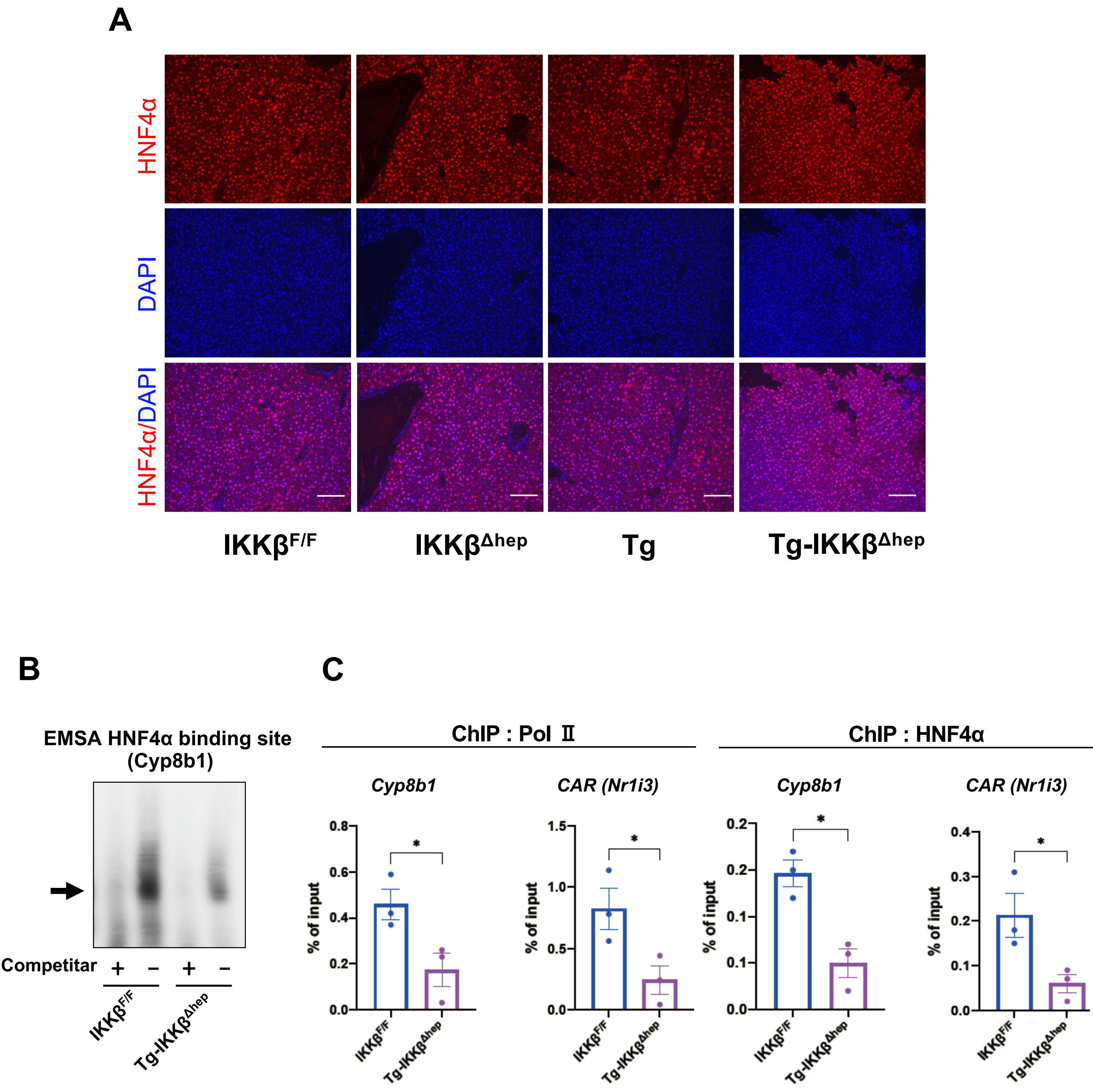

Supplementary Figure S10

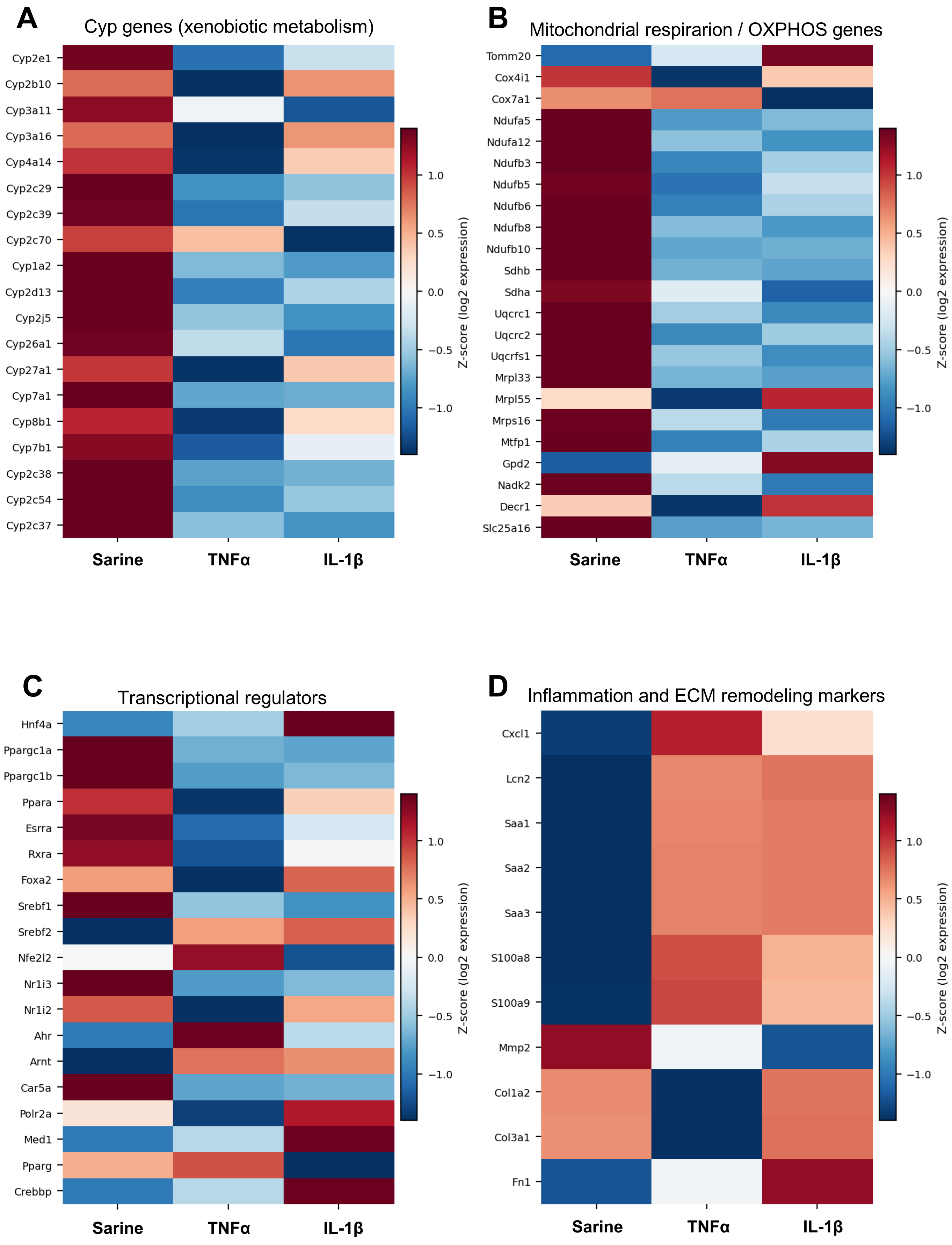

Supplementary Figure S11

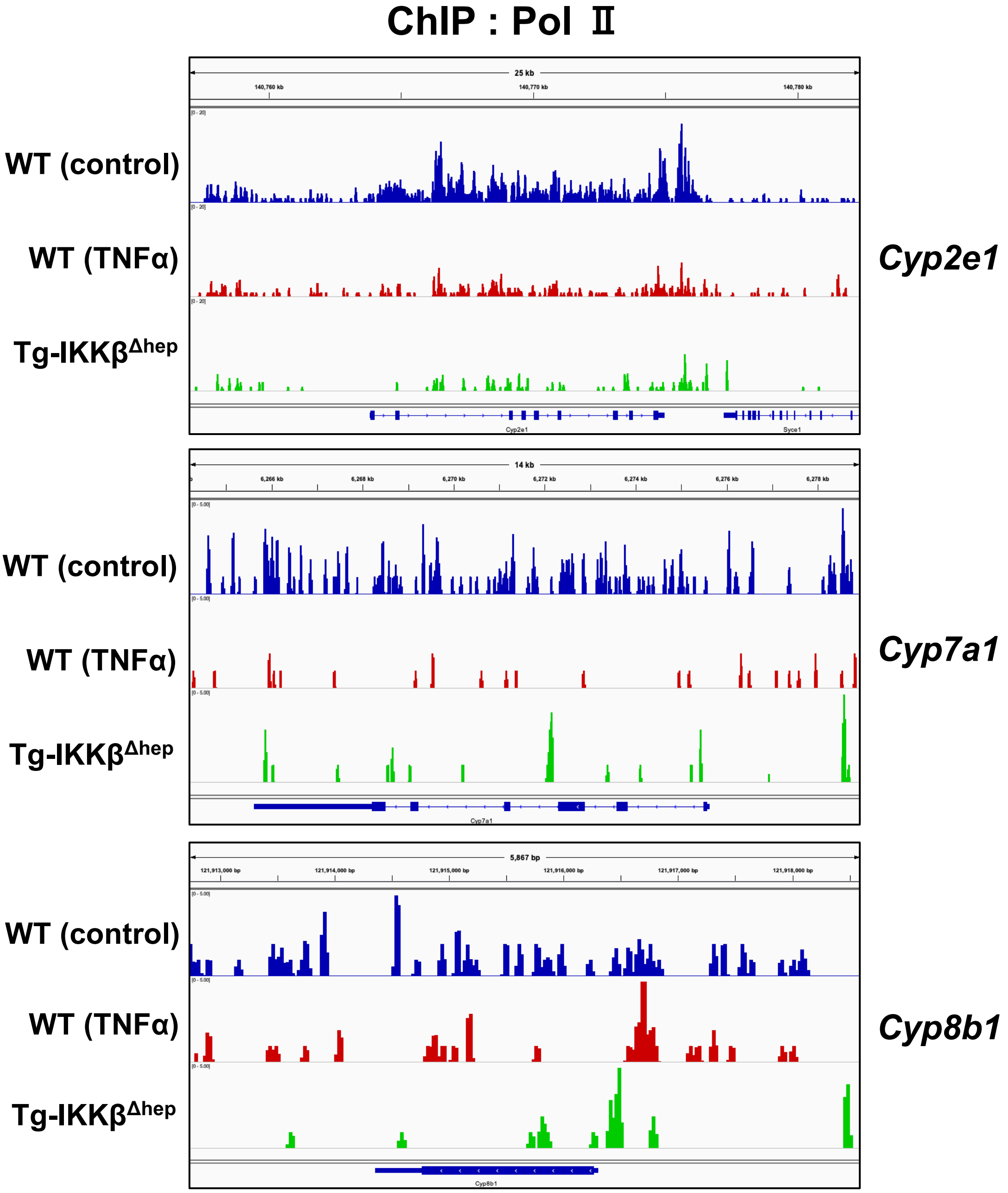

Supplement: Supplementary file 1 [file cells-15-00546-s001.zip › Supplementary Figure S1-S11 final.pdf]
