# Supplementary material for: Inflammation-Driven Downregulation of CYP2E1 Is Associated with Attenuated Diethylnitrosamine (DEN)-Induced Hepatocarcinogenesis
Source: Cells. 2026 Mar 19;15(6):546. doi: 10.3390/cells15060546 (PMC13025445; doi:10.3390/cells15060546)
Supplement: Supplementary file 1 [file cells-15-00546-s001.zip › Supplementary Table S1.pdf]

Table S1. Genotyping primers used in this study.

Primer sequences are shown in the 5′→3′ direction.

**Target (allele/transgene)**	**Primer**	**Sequence (5′→3′)**
Cre recombinase transgene	Cre-F	ACCTGAAGATGTTCGCGATTATCT
Cre recombinase transgene	Cre-R	ACCGTCAGTACGTGAGATATCTT
Ikkβ/Ikbk2 floxed allele	IKKβ-lox-F	GTCATTTCCACAGCCCTGTGA
Ikkβ/Ikbk2 floxed allele	IKKβ-lox-R	CCTTGTCCTATAGAAGCACAAC
Ikkβ exon 3 deletion (IkkβΔ) confirmation	IKK-74F	TAGTCCAACTGGCAGCGAATAC
Ikkβ exon 3 deletion (IkkβΔ) confirmation	IKK-188R	CGCCTAGGTAAGATGGCTGTCT
FLAG-tagged transgene (e.g., FLAG-NLS-IKKβ)	FLAG-F	GACTACAAGGACGACGATGA
FLAG-tagged transgene (e.g., FLAG-NLS-IKKβ)	IKK-R	CATGTCTGCGTTGTCAGGGA
