# Supplementary material for: Inflammation-Driven Downregulation of CYP2E1 Is Associated with Attenuated Diethylnitrosamine (DEN)-Induced Hepatocarcinogenesis
Source: Cells. 2026 Mar 19;15(6):546. doi: 10.3390/cells15060546 (PMC13025445; doi:10.3390/cells15060546)
Supplement: Supplementary file 1 [file cells-15-00546-s001.zip › Supplementary Table S2.pdf]

Table S2. Primer sequences used for RT-qPCR (grouped by functional).

All primer sequences are shown 5′–3′direction.

Target	Forward primer (5′–3′)	Reverse primer (5′–3′)
**Metabolic regulation**		
PPARα	ATGCCAGTACTGCCGTTT	GGCCTTGACCTTGTTCATGT
**Metabolism**		
CYP2C54	AGACAGAGCTATGAAAGAGGGAA	GTGAGAAGTGCCTCGTGTTTT
**DEN metabolism**		
CYP2E1	CGTTGCCTTGCTTGTCTGGA	AAGAAAGGAATTGGGAAAGGTCC
**Bile acid synthesis**		
CYP7A1	CACTCTACACCTTGAGGATGG	GACATATTGTAGCTCCTGATCC
CYP8B1	CCTCTGGACAAGGGTTTTGTG	GCACCGTGAAGACATCCCC
**Fibrosis marker**		
Collagen 1a1	GAGCGGAGAGTACTGGATCG	GCTTCTTTTCCTTGGGGTTC
TIMP-1	ATTCAAGGCTGTGGGAAATG	CTCAGAGTACGCCAGGGAAC
α-SMA (Acta2)	ACCAACTGGGACGACATGGAA	TGTCAGCAGTGTCGGATGCTC
**ECM remodeling**		
MMP-9	AACCTCCAACCTCACGGACA	TCCCACTTGAGGCCTTTGAA
**Coagulation regulator**		
PAI-1	TTCAGCCCTTGCTTGCCTC	ACACTTTTACTCCGAAGTCGGT
**Inflammation marker**		
TNFα	GAACTGGCAGAAGAGGCACT	AGGGTCTGGGCCATAGAACT
**Inflammatory cytokine**		
IL-1β	AGGAGAACCAAGCAACGACA	TGCTTGTGAGGTGCTGATGT
IL-6	CCATCCAGTTGCCTTCTTGG	TCCACGATTTCCCAGAGAACA
**Nuclear receptor**		
HNF4α	ATTGCCAACATCACAGACG	GTAATCCTCCAGGCTCACTT
PXR (Nr1i2)	GATGGAGGTCTTCAAATCTGCC	GGCCCTTCTGAAAAACCCCT
CAR (Nr1i3)	GGAGCGGCTGTGGAAATATTGCAT	TCCATCTTGTAGCAAAGAGGCCCA
**Mitochondrial biogenesis**		
PGC1α (Ppargc1a)	TATGGAGTGACATAGAGTGTGCT	CCACTTCAATCCACCCAGAAAG
**Cell cycle regulator**		
p21 (Cdkn1a)	ATGTCCAATCCTGGTGATGT	TGCAGCAGGGCAGAGGAAGT
**Housekeeping gene (cyclophilin)**		
CPH	ATGGTCAACCCCACCGTGT	TTCTTGCTGTCTTTGGAACTTTGTC
**Apoptosis regulator**		
Bax	ATGCGTCCACCAAGAAGCTGA	AGCAATCATCCTCTGCAGCTC
