# Supplementary material for: Inflammation-Driven Downregulation of CYP2E1 Is Associated with Attenuated Diethylnitrosamine (DEN)-Induced Hepatocarcinogenesis
Source: Cells. 2026 Mar 19;15(6):546. doi: 10.3390/cells15060546 (PMC13025445; doi:10.3390/cells15060546)
Supplement: Supplementary file 1 [file cells-15-00546-s001.zip › Supplementary Table S3.pdf]

Supplementary Table S3. Oligonucleotide sequences used for EMSA and ChIP–qPCR.

All sequences are shown in the 5′→3′ direction.

Assay	Target	Oligonucleotide	Sequence (5′→3′)
EMSA	Cyp8b1 promoter (HNF4α-binding site)	Probe-F	GCGTCCGAGCCTCTGAGCAAAGTCCAAGGGCA
EMSA	Cyp8b1 promoter (HNF4α-binding site)	Probe-R	GGTTCCTGCCCTTGGACTTTGCTCAGAGGCTCG
ChIP–qPCR	Gapdh promoter	Forward	TGCACGTAGCTCAGGCCTCTGCGC
ChIP–qPCR	Gapdh promoter	Reverse	ACCTGGCACTGCACAAGAAGATG
ChIP–qPCR	Cyp2e1 promoter	Forward	GTCAGATCAGTAGATGCAAC
ChIP–qPCR	Cyp2e1 promoter	Reverse	TAGGAATTTCCTGAGGAGAA
ChIP–qPCR	Cyp7a1 promoter	Forward	AGGGACAGACCTTCGGCTTA
ChIP–qPCR	Cyp7a1 promoter	Reverse	TGGGTGACCAGAGCAAACAC
ChIP–qPCR	Cyp8b1 promoter	Forward	AGGCAGGCAACCATGGAGAT
ChIP–qPCR	Cyp8b1 promoter	Reverse	GTTCCTGCCCTTGGACTTTG
ChIP–qPCR	Car (Nr1i3) promoter	Forward	CTCACCCACAGGGAAATCCTCC
ChIP–qPCR	Car (Nr1i3) promoter	Reverse	CTGACCCTGCTTTCCTTGAGATC
